# Dynamic Routings in Satellite Networks: An Overview

**DOI:** 10.3390/s22124552

**Published:** 2022-06-16

**Authors:** Xiaoli Cao, Yitao Li, Xingzhong Xiong, Jun Wang

**Affiliations:** Artificial Intelligence Key Laboratory of Sichuan Province, School of Automation and Information Engineering, Sichuan University of Science and Engineering, Yibin 644000, China; 320081104115@stu.suse.edu.cn (X.C.); xzxiong@suse.edu.cn (X.X.); 320085404111@stu.suse.edu.cn (J.W.)

**Keywords:** single-layer satellite networks, multi-layer satellite networks, dynamic routings, overview

## Abstract

The Satellite network is an important part of the global network. However, the complex architecture, changeable constellation topology, and frequent inter-satellite connection switching problems bring great challenges to the routing designs of satellite networks, making the study of the routing methods in satellite networks a research hotspot. Therefore, this paper investigates the latest existing routing works to tackle the dynamic routing problems in satellite networks. The architecture and development of satellite networks are presented and analyzed first. Afterward, dynamic routing problems in satellite networks are analyzed in detail based on the time-varying network topology. According to the latest works, the advanced satellite network routing schemes, including single-layer and multi-layer dynamic routing are introduced and analyzed. In addition, the merits, shortcomings, and applications of these schemes are analyzed and summarized. Finally, potential technologies and future directions are discussed.

## 1. Introduction

In traditional terrestrial networks, routers calculate routing tables from a routing information database established from network topology information. While updating the routing information base relies mainly on changing the link-state, such routing updates require the entire network to share topology information, resulting in greater network overhead, slow convergence, and other drawbacks of the routing protocols [1]. Since the topology of the terrestrial network varies less frequently, updating the routing table is no longer necessary once the network reaches a stable state. Meanwhile, the computing power and storage capacity of the router are sufficient to meet the calculation requirements of the routing table, so the existing routing protocols can be better adapted to the terrestrial network.

Compared to terrestrial networks, satellite networks can provide global coverage and efficient communication services without the constraints of geography and infrastructure [2,3]. The satellite network topology is highly dynamic and time-varying, and satellites have limited onboard computing and storage due to their size and power consumption. In particular, the relatively long inter-satellite distance and link transmission delay are the crucial factors affecting the routing performance of satellite networks [4]. Additionally, terrestrial network routing algorithms are not directly applicable to satellite networks due to their high complexity and processing requirements [5,6]. Therefore, both academia and industry must develop new routing algorithms depending on the characteristics of the satellite network itself [7]. This paper provides a brief overview of the current dynamicrouting algorithms available in the satellite networks and discusses potential techniques and research directions.

The routing algorithm must be capable of adapting to the dynamic satellite network topology and sensing changes such as network load traffic changes and link congestion states in a timely manner. In light of this, this paper proposes solutions for single-layer satellite network routing algorithms from different perspectives. However, the performance of a multi-layer satellite network comprised of low-Earth orbit (LEO), medium-Earth orbit (MEO), and Geosynchronous Earth orbit (GEO) is superior to that of a single-layer satellite network. Therefore, this paper presents the most recent algorithm for dynamic routing in multi-layer satellite networks. These algorithms can solve link congestion and load traffic balancing problems and adjust quality of services (QoS) parameters based on network conditions. In addition to having a high throughput, this class of algorithms guarantees a reasonable end-to-end delay. The contributions of this paper are summarized as follows:

- The satellite networks are introduced in this paper, and the merits and drawbacks of the LEO, MEO, and GEO networks are discussed in this paper. The issues of dynamic routings in satellite networks are analyzed according to the dynamic network topology.
- The latest satellite network routing works are summarized, including single-layer and multi-layer dynamic routings. Meanwhile, the applicability and virtues in solving the problem of satellite networks are analyzed in this paper.
- Potential technologies and future directions in dynamic satellite routings are provided and discussed, including machine learning, mobile edge Computing, digital twin, multiscale information awareness and computing in a complex environment, intelligent satellite, and intelligent routing.

**Figure 1:**
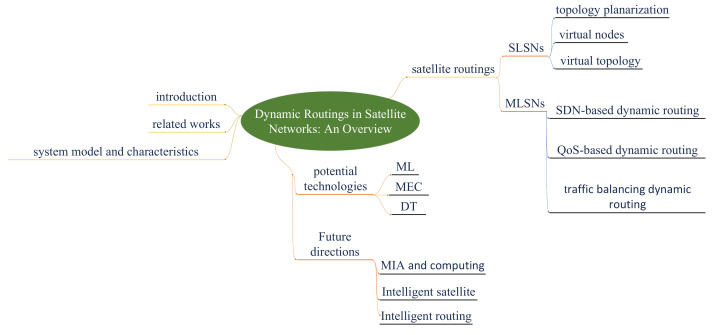
Article layout.

The remainder of this paper is structured as follows: Section 2 briefly describes the model, characteristics, and several key technologies of satellite networks. Four hotspots in satellite routing technologies are introduced emphatically in Section 3. Section 4 prospects future existing work directions and potential routing technologies. Finally, conclusions are in drawn Section 5. The structure diagram of the paper is shown in Figure 1.

## 2. Related Works

There have been many existing works on satellite routings published in recent years. The majority of the existing works on routing problems in satellite systems analyze routing models and routing strategies. The current achievements of satellite routings have been analyzed from various angles.

The work in [8] investigated the space–air–ground integrated network, with a special focus on two kinds of IP routing algorithms: traffic-based algorithms and QoS-based algorithms. Given that SAGIN’s services have distinct QoS requirements, assigning their service flows to the proper terrestrial, satellite, or air-to-ground links based on QoS requirements and link quality is worthwhile. Therefore, the authors analyzed the traffic-based routing algorithm. The algorithm considers three classes of traffic: (i) delay-sensitive, (ii) throughput-sensitive, and (iii) best-effort. In order to achieve better performance in avoiding congestion, reducing queueing delay, lowering packet drops, and increasing total throughput, a traffic-light-based intelligent routing (TLR) scheme for satellite internet protocol (IP) networks was developed. Traffic lights are used to indicate the congestion status at both the current node and the next node. With the growth of mobile data traffic in future terrestrial 6G wireless networks, satellite capacity is being used to reduce traffic and lighten the stress on terrestrial links. The UAV-assisted air–ground communication distance can be significantly reduced, and the capacity of the network can be increased by appropriate routing algorithms. Hence, a connectivity-based traffic-density-aware routing algorithm for vehicular and ad hoc networks (VANETs) employing uncrewed aerial vehicles (UAVs) has been proposed. By using UAVs as the relay nodes and combining the real-time traffic density based on the periodic exchange of messages, the algorithm could find the shortest stable connected path to forward packets to their destinations at each moment. In addition, the authors analyzed a multi-path QoS routing algorithm based on a polar-orbiting satellite network. They provided insight into the impact of some key factors on the observed QoS parameters. The QoS metrics mainly include end-to-end delay, delay jitter, and bandwidth in this algorithm. However, due to the high mobility of LEO satellites, some ISLs in the network are not always available. Therefore, there should be additional QoS elements to measure the network performance. The authors changed the genetic algorithm’s fitness function, variation probability based on satellite congestion, and simulated annealing to handle population variety. Finally, an inter HAPs-satellite routing method is investigated to maximize satellite link capacity and achieve network load balancing. Simulations demonstrate that the algorithm can achieve the same throughput while saving 30% of the satellite’s link capacity.

The work in [9] reviewed the routing algorithms in satellite networks. Based on virtual nodes, the authors focus on three routing algorithms. First, the localized zone distributed routing (LZDR) scheme was analyzed. The routing process of the algorithm was divided into two parts: inter-zone and intra-zone routing. Finally, the low-complexity probability routing algorithm (LCPR) was used to solve the next-hop selection when the satellite received data packets. However, the LZDR algorithm only described the method of computing inter-domain routing based on the path with minimum hops. In addition, the LZDR algorithm is only suitable for the polar orbit of LEO satellite networks, not for the tilted orbit satellite constellations. The driven routing algorithm (DRA) derives the constellation topology from the bound ground base station. When satellite nodes or links fail, the performance of the DRA algorithm is significantly degraded. The LCPR is used to solve next-hop selection when a satellite receives a packet. By utilizing the position message of the source node and destination node, the distributed computing method is used to obtain the optimal path. Each node can dynamically select the next-hop by informing its neighbors of the congestion state in the algorithm. Thus, the average queue time delay and packet loss rate are decreased.

For multi-layer satellite networks, the authors focused on four routing algorithms. First, the authors analyzed the satellite grouping and routing protocol (SGRP). The main idea of SGRP is to transmit packets through the shortest delay path. SGRP includes three phases: delay reporting from LEO satellites to the MEO layer, delay exchange at the MEO layer, and routing table construction. In addition, the SGRP routing algorithm provides a mechanism to resolve congestion and satellite failures to avoid packet loss. Then, the authors analyzed the hierarchical satellite routing protocol (HSRP). HSRP applies to satellite-to-satellite (SOS) networks for long-distance correlation transmission. SOS is a combined network with multi-layer constellations. In SOS networks, the orbital altitude and number of layers can vary depending on the performance and type of services provided. HSRP identifies paths that meet delay constraints and improves resource efficiency. To make the satellite network autonomous, the authors analyzed the SARA algorithm. SARA partitions the network topology and calculates the routing table using ISL’s connection criteria. When a node fails, the GEO satellite can recalculate the routing table for the failed area. Finally, the authors analyzed the expanding range route selection (ERRS) routing algorithm. ERRS can find the EDT optimal route by searching each snapshot of the time-varying topology. Simulations demonstrate that the algorithm can cut the end-to-end transmission time and boost the network throughput. Additionally, the author also summarized routing methods in multi-layer satellite networks [10]. Firstly, the satellite group and group management (SGGM) routing were used to implement a three-layer network structure suitable for MEO/LEO two-layer satellite networks. Then, the disruption-ant network (DTN) was analyzed. Afterward, the data-driven routing algorithm (DDRA) was analyzed for the GEO/LEO two-layer satellite networks. Finally, based on region partition, an improved short hopping path was used to balance the network traffic and reduce the queue delay. Although these algorithms could provide optimal selection and dynamic adaptability, they would increase the overhead of routing protocols between satellite layer groups.

The LEO satellite network, as an essential part of satellite communication network, is one of the hot spots of current research. The dynamic virtual topology routing (DVTR) algorithm introduced a two-phase routing algorithm to compute the virtual path table. The footprint handover rerouting protocol (FHRP) was analyzed to reduce the overhead of updating the routing table. FHRP was a connection-oriented routing algorithm designed to reduce network overhead caused by frequent routing table updates. Although the above approaches can balance network traffic, this type of routing algorithm cannot respond to both link load changes and link-state changes [11]. Nevertheless, the routing performance was significantly degraded when the network topology lost its regularity. Uneven terrestrial service distribution may lead to inter-satellite link congestion (LRES), making load balancing a key issue for the LEO satellite network. Therefore, by extending the range of available paths, combined with congestion avoidance mechanisms, a load balancing routing algorithm based on extended link states was analyzed in [12]. The scheme maintains path optimality and decreases network congestion. In the algorithm, the network consists of inter-satellite links (ISLs) with LEO satellites. In order to enable better data transmission in this network, the designed routing algorithm can dynamically adjust the path according to the link state in the network. At the same time, LEO satellites are source and relay nodes, so the routing algorithm must respond to load and link-state changes. Simulation findings show that the approach balances the service load, decreases link congestion and packet loss, and enhances the LEO satellite network’s throughput. In conclusion, the algorithm outperformed ELB and TLR algorithms in terms of effective network traffic load balancing. In particular, the algorithm improves LEO network performance by reducing packet loss and increasing throughput under load. In addition, the authors also investigated a connection congestion notification and rerouting strategy. A comparison of the partial routing algorithms mentioned is shown in Table 1.

The work in [13] provided a detailed description of the architecture of the space–air–ground integrated network (SAGIN) and immersive media (IM) services. The literature on the SAGIN architecture was explicitly introduced, and its architecture, advantages, and critical indices were reviewed. The SAGIN literature review is divided into three sections. The authors investigated resource allocation and communication methods in diverse networks in the first category. Second are the SDN-based SAGIN designs for intelligent management and orchestration. Third are the SAGIN architectures in specific application contexts. IM is an emerging service model whose virtual nature has attracted the attention of most scholars. However, IM has the drawbacks of having excessive bandwidth, a low transmission rate, and high latency regarding network performance. Then, to address the issue, the authors proposed the service customized SAGIN architecture for IM (SAGIM) based on the Service customized network (SCN). The integrated network is the core component of SAGIM. It consists of a space segment, an air segment, and a ground segment. The space segment includes GEO and LEO satellites. They communicate with each other via microwave or laser. The LEO satellites support broadband Internet access for IM services. The solution further designed the functional components of SAG-IM, including the infrastructure layer, sensing layer, intelligence layer, and application layer, to enable intelligent network management. As the core foundation of the SAG-IM architecture, the infrastructure layer provides the fundamental guarantee for the operation of the integrated network. The network infrastructure includes the network entities and links that ensure the robustness of the network. To separate services for different users, hardware resources are divided into communication, computing, and storage resources. These virtual resources provide data processing and transmission for different services in the network. The sensing layer is used to collect the network state of the infrastructure layer, including node and link information, etc. The intelligence layer is the brain of the SAGIM architecture and is responsible for scheduling network resources. In addition, the Intelligent layer load establishes routing and forwarding strategies to assure QoS for other IM requests. The application layer ensures SAGIM’s efficient functioning and flexible network setup. In particular, to improve the intelligent management of the network, the processing of the application layer is outlined in detail. The authors also analyzed the clustering technology of multi-tier SDN controllers and the deployment technology of edge servers. The layered SDN controller deployment policy implements intelligent routing, throughput control, load balancing, etc. SAGIM controllers are placed in ground data centers and satellites, improving the update and configuration performance. As SAGIN has some limitations in instant messaging services, to enhance the user experience, key technologies for intelligent routing and delivery were further discussed in the paper. Finally, the author analyzed and summarized the research directions and potential technical challenges of SAGIM in future applications.

The space–air–ground–sea integrated network (SAGSIN) combines satellite, ground, air, and maritime networks for 6G communication. With its diverse network composition, open communication environment, and time-varying network topology, the SAGSIN encounters severe threats to its security. However, the safety issues of the SAGSIN are not thoroughly studied by domestic and foreign scholars. Therefore, the work in [14] presented a unified summary of the research work related to SAGSIN security. First, the article investigated the current status of SAGSIN and the network architecture system, as well as its characteristics and technical difficulties. Next, the security requirements of the SAGSIN system were discussed, focusing on various types of representative networks. Then, the authors provided a detailed description of the security threats, attack methods, and defense strategies of SAGSIN systems. Moreover, the security problems, attacks, and defense methods of SAGSINs were highlighted, including multi-layered and diverse network structures. This paper explores security precautions such as anti-interference techniques, secure routing algorithms, secure switching mechanisms, key management mechanisms, and IDSs to address interference, eavesdropping, denial of service, and fraud attacks in SAGSIN systems. Finally, the article outlined cross-level attacks and security precautions in the SAGSIN system, pointing out possible new challenges and future trends in research.

As digital connectivity grows globally, major network components must be rebuilt and reassessed. Based on the giant LEO constellation, the work in [15] introduced the application of the ground network in the next-generation wireless communication network. While the strengths of the LEO satellite network can compensate for terrestrial network weaknesses, combining the two can significantly impact network design and deployment. Then, the paper presented a systematic study of the future development of large LEO satellites and summarized the constellation network’s favorable conditions and major problems. Additionally, a comprehensive review of the large LEO satellite network was conducted to realize its performance simulation better. Simulation software can accurately characterize the satellite network’s operation. Special network details require extensive iterative operations in the simulation algorithm. The simulation system is unique because its special structure cannot be extended to other networks. Deploying specific analytical models during the initial development process can consider satellite altitude, the number of satellites in the constellation, orbital planes, and other critical network factors. Finally, the analytical model is significant for other large and complex satellite networks.

Satellite communications offer lower latency and faster transmissions due to the growth of LEO satellite networks. However, the time-varying topology of the entire network makes the routing problem of the LEO satellite network challenging. Inspired by machine learning approaches to network routing problems, several researchers have studied the LEO satellite routing problem in reinforcement learning [16]. However, in current research, multi-step learning has rarely been studied to speed up training and improve learning efficiency. Therefore, the work in [17] proposed a routing scheme based on deep reinforcement learning for satellite routing (DRL-SR). This scheme aims to assign the corresponding destinations to all users’ routing requests in a DTN-based satellite network. The scheme focuses on two factors: delay and link capacity. The authors suggested a non-deterministic polynomial-time hard (NP-hard) pure binary integer programming optimization approach. The authors reconstructed the model using the Markov decision process (MDP) to improve the learning rate and quality. Based on this model, the authors proposed a multi-depth Q-network (DQN)-based RL method. The solution uses multi-step learning to route user requests efficiently through the agent. In addition, the authors utilize greedy strategies to allow the agent to explore new surroundings and make numerous routing decisions for user requests. In numerical experiments, the authors evaluated the proposed scheme’s performance through network delay with varying numbers of satellites and users. The simulation results show that the authors’ proposed DRL-SR learning algorithm outperforms the shortest path algorithm.

Current maritime communications rely mainly on satellites with weak transmission resources and lesser performance than modern terrestrial wireless networks. To meet heterogeneous business requirements, the work in [18] proposed a SAGIN framework. However, due to heterogeneity, self-organization, and time unpredictability, designing and optimizing the SAGIN framework is challenging. The primary difficulty is designing an effective routing system that can handle a highly dynamic network architecture. Recent advances in artificial intelligence have generated various routing algorithms for wireless communications, such as deep learning (DL)-assisted routing algorithms for balancing traffic in SAGINs [19]. However, in such routing algorithms, the network topology is assumed to be static, and routing decisions are made based on the state of the nodes in the global network. Therefore, to handle highly dynamic network topologies, the authors designed a deep reinforcement learning (DRL)-assisted routing algorithm for ad hoc aeronautical networking (AANET). The algorithm relies entirely on local information and can achieve near-optimal end-to-end delays. To meet the needs of heterogeneous services and to accommodate the dynamic nature of SAGINs, the authors further propose a DL-assisted multi-objective routing algorithm. The algorithm utilizes a quasi-predictable network topology and operates in a distributed manner. The author proposed a DL-assisted routing algorithm that minimizes end-to-end delay to aid comprehension. For single destination routing, each snapshot of the network topology can calculate link delays based on each node’s coordinates and delay model. The approach trains a single-objective deep neural network (SO-DNN) to embed network topology information. During algorithm training, all nodes’ queuing delays are constant. Then, the SO-DNN calculates the minimal delay between each source–destination pair using the shortest path technique. The authors also utilized local information to solve the multi-target routing problem for multi-target routing. Therefore, similar to SO-DNN, the algorithm uses a multi-objective deep neural network (MO-DNN) to learn. Experimental results show that the integrated network achieves better network coverage, lower latency, higher throughput, and a longer path life.

Due to the highly dynamic and time-varying nature of the topology of satellite networks. Based on the characteristics of satellite networks, academia and industry must develop new routing algorithms to improve the stability of satellite routing algorithms and satellite communication performance. However, the above overview works did not focus on the uniqueness and new key issues of dynamic routing in satellite networks in timely manner, especially for the requirements, challenges, and solutions of dynamic routings in large-scale multi-layer satellite networks. The comparison of related works is shown in Table 2.

## 3. System Model and Characteristics

Satellite networks are an essential part of the space–air–ground integrated network. They not only provide coverage for remote and off-land regions, but they also provide emergency communications. It is crucial for seamless global transmissions, the global Internet of Things, and emergency communications.

### 3.1. System Model

**Figure 2:**
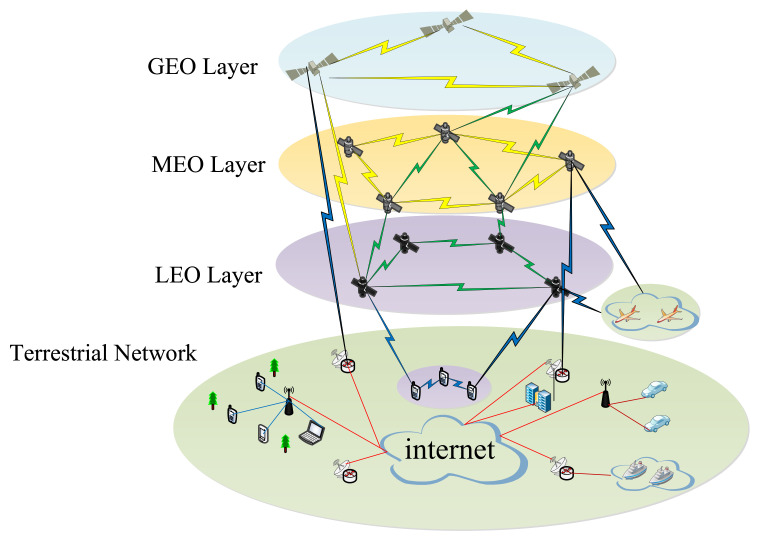
Space–air–ground integrated network.

The space–air–ground integrated network has become the inevitable trend of the future network. It consists of a satellite network, an air network of various flying probes, and aground network. A model of the space–air–ground integrated network is shown in Figure 2.

Since they are highly dynamic network environments, most existing space–air–ground integrated network routing strategies are insufficient for interactive information transmission between heterogeneous layers. In particular, satellite networks are characterized by high dynamics. The satellite networks are constructed by integrating several satellite networks and hierarchical structures. The satellite networks can be divided into single and multi-layer satellite networks. The single-layer satellite network consists of one or more orbital planes. Each satellite is generally equipped with an inter-satellite link to communicate with neighboring satellites. Meanwhile, the satellite can interact with the ground gateway station and the user station via the feeder and user links, creating a complex communication system with multiple links.

The multi-layer satellite networks are usually composed of two or more satellites with different orbits. Different altitudes of satellites have different effects on service performance. In addition, there are many various types of applications, and each has its QoS. Due to the quick movement of different satellites, dynamic routing is one of the most fundamental issues for multi-layer satellite networks. Many existing multi-layer satellite networks work mainly on GEO/LEO or MEO/LEO two-layer satellite networks and GEO/MEO/LEO three-layer satellite networks. Table 3 shows a comparison of the characteristics of the GEO/LEO or MEO/LEO two-layer satellite networks and the GEO/MEO/LEO three-layer satellite network.

### 3.2. Network Characteristic

In the satellite networks, the relative movement of the satellite nodes has certain regularity, and the positions are not fixed as the general ground nodes. The change in link quality caused by the relative motion of satellite nodes is an urgent problem in space networks. Because of the frequent changes in links, network management becomes over-dependent on time, and the whole network topology plans will be influenced. The following are the main features of the satellite network: (a) *broadcast mode operation allows for multi-access communication;* (b) *large communication capacity for multiple service transmission;* (c) *the ability to compose complex network topologies;* (d) *safety and reliability.* Traditional routing strategies in spatial networks have many problems, such as the frequent handover of links, the high routing overhead, and the poor quality of service.

Since the dynamic satellite topology structure is predictable, the centralized control scheme based on software-defined network (SDN) technologies is adopted to design the routing mechanism. The ground SDN controller completes the routing calculation. By combining inter-satellite and terrestrial user links, a private multiprotocol label switching (MPLS) protocol can carry and exchange multi-layer network protocols. The ground station SDN controller handles routing protocols such as open shortest path first (OSPF); configures network address mapping tables for tags, satellites, and terminals; and deletes MPLS and label distribution protocol (LDP) switching paths.

### 3.3. Key Challenges for Satellite Network Routing

Routing technology has been the focus of research in satellite networks. The earlier satellite networks generally used GEO satellites as relays and bent tube transponders for data forwarding between two stations on the ground. Such a data transfer form is fixed, and no routes are available. In a constellation network with many satellites, the satellite network must select the optimal path among multiple reachable paths between the source and destination satellite nodes. Therefore, several challenges are still faced for satellite routing, mainly in the following aspects.

Link Switching on Routing: Due to the rapid movement of satellites, specific switching mechanisms are needed to maintain communication continuity. However, inappropriate link switching may cause re-routing issues in the satellite networks. Re-routing can generally be separated into partial re-routing and complete re-routing.The partial re-route scheme refers to re-routing only the links on which the switchover occurs based on the current network state and routing situation. Although such routing is relatively simple, the route selected utilizing the routing mechanism is not guaranteed to be the optimal route. Complete re-routing means redistributing new routing paths for communicating with users after the link has already completed switching. For such routing strategies, the optimal routing of the network is ensured but at the expense of a certain computational overhead. Therefore, to resolve the re-routing problem and improve the routing performance of satellite networks, more profound studies on link switching strategies are needed in academia and industry.

High-Transmission Delay and Channel BER:In satellite networks, owing to the long-distance transmission between satellites of different layers, the latency of transmission across inter-satellite links will be considerably higher than the nodes in terrestrial networks. The link transmission delay between LEO satellites is 15–25 ms, while the transmission delay between MEO satellites is 40 to 60 ms. As the physical conditions of space networks are affected, noise greatly interferes with the modulation and error correction of communication signals during inter-satellite links, resulting in a much higher channel BER of satellite networks than terrestrial networks. Although such noise is less disruptive to the laser link, the link still requires a high level of satellite attitude control. In the communication process, the satellite attitude control will affect the communication quality of the satellite network. However, when calculating satellite routes with the dynamic routing algorithm onboard, it is extremely challenging to fully reflect the real-time state between satellite links through network state updates alone. Furthermore, the approach is prone to making each satellite node calculate routing tables with inaccurate network state information, leading to the waste of on-star computing resources.

Restricted on-star Processing Power and Storage Resources: Constraints include the spatial environment and satellite loading technology, such as high ionizing radiation and low power consumption. The processing power and storage resources on the satellite are extremely limited. Hence, storing all the status information of the whole network on the star is impractical. Owing to the highly dynamic topology of the satellite network, obtaining link status information from the ground network system alone cannot guarantee accurate results. With the limited on-star computing power, the on-star routing algorithm must have low implementation complexity (namely, low computational complexity), communication costs, and storage costs. In addition, owing to the unique launch mode of the satellite, updating and upgrading its functions is hardly possible.

Highly Dynamic Network Topology: LEO and MEO satellites operate at high speeds, resulting in a highly dynamic network topology. On the one hand, due to fading signal and ground obscuration, the satellite and user can only communicate at a high elevation. Since the LEO satellite has a large orbital altitude and pitch angle, the area covered on the surface is somewhat limited. On the other hand, the LEO satellite moves very fast, causing its coverage on the surface to change quickly as well. Consequently, the user terminal will be switched between LEO satellites, i.e., user-to-sat switching. Similarly, a multi-connector link generally passes through more than one ISL. The rapid movement of satellites can cause the failure of one or more ISLs in high latitudes. Under such a situation, a satellite-to-satellite switch, or sat-to-sat switching, occurs. For time-varying satellite networks, none of the switches can cause the established routes not to work correctly. Re-routing mechanisms increase network latency and cannot meet QoS latency requirements, causing communication disruptions.

Unbalanced Load Traffic: Geographical conditions, satellite motion, and the Earth’s rotation characterize the time-varying nature and uneven distribution of load traffic in satellite networks. A single satellite can cover a small populated area, such as the Arctic, and a densely populated area, such as a developed country. As the satellite is constantly in motion, the number of terminal users and the amount of load traffic within the network are changing. Since there is not enough resource space for up/down link (UDL) routing between satellite users, variations in such load traffic may affect some switching strategies in the network. For one, even if the ISL has sufficient resource capacity at the time of establishment, the change in link load traffic can cause network congestion, preventing the link from switching. In addition, even under the same user load traffic conditions, each LEO satellite and ISL may have different load traffic, causing link switching to fail. The blocked link prevents QoS routing, affecting satellite network communication.

### 3.4. Key Technologies

(1) Inter-Satellite High-Speed Transmission Technologies: Due to the high transmission rate and small terminal volume, inter-satellite laser technologies have become the leading choice for constructing inter-satellite links. Microwave signals can be modulated on an optical carrier by frequency modulation. There are two corresponding reception nodes: direct intensity detection and coherent detection. The technical scheme of intensity modulation and direct detection (IM/DD) is economical and straightforward, but it is easy to introduce noise. The modulation frequency is low, and the receiving sensitivity is relatively poor. Terahertz communication has a widely available spectrum and simple beam-tracking, making it one of the most important future technologies for high-speed transmission and networking in space.

(2) Dynamic Routing Technologies: The movement of LEO satellites makes the topology of the whole network change constantly, and the frequent link handoffs between satellites bring new challenges to the design of spatial routing protocols [20]. A routing algorithm based on a snapshot sequence is a mature algorithm in the satellite constellation. The algorithm divides the topology of satellite networks into several individual snapshots, and the constellation topology in the snapshot is stable and predictable. Therefore, we are able to precompute the routing table in each snapshot and continue switching.

(3) Onboard Processing Technologies: Onboard processing (OBP), onboard switching (OBS), and onboard routing (OBR) are among the technologies. In order to realize the constellation broadband system, effectively connect to the terrestrial broadband network, and meet the service level and quality of service requirements of the user, onboard processing technologies are crucial. Onboard processing technologies are used to realize the upgrading of hardware and software technologies. The hardware technologies mainly include surface acoustic wave (SAW) filtering channelization, fast switching technologies, etc. Software technologies include virtual nodes, cross-layer resource allocation, and scheduling algorithms.

(4) Inter-Stellar Link: Inter-stellar link (ISL) is the foundation of constellation communication, and connecting satellite nodes. With the development of science and technology, laser communication technology is gradually being applied in inter-stellar link communication, resulting in a significant increase in ISL bandwidth. The laser reduces the communication delay significantly. However, the ISL of laser communication has stringent requirements for satellite attitude control. The slight instability of satellite attitudes will result in a disruption in the communication.

(5) Dynamic Network Slicing: The motivation of network slicing in a space information networks (SIN) is to allocate as few resources as possible to satisfy the end-to-end transmission demands. Theresource requirements vary with the types of services. For delay-sensitive services, such as video communication, high reliability and low delay paths are required to support the real-time transmission, but the delay-tolerant services, such as Earth observation, require a high data rate with a tolerant time delay. Therefore, for the different types of services, accurately acquiring the status of the available resources and the flexible scheduling of the multi-dimensional resources are the core issues of dynamic network slice design.

## 4. Existing Works on Dynamic Satellite Routings

**Figure 3:**
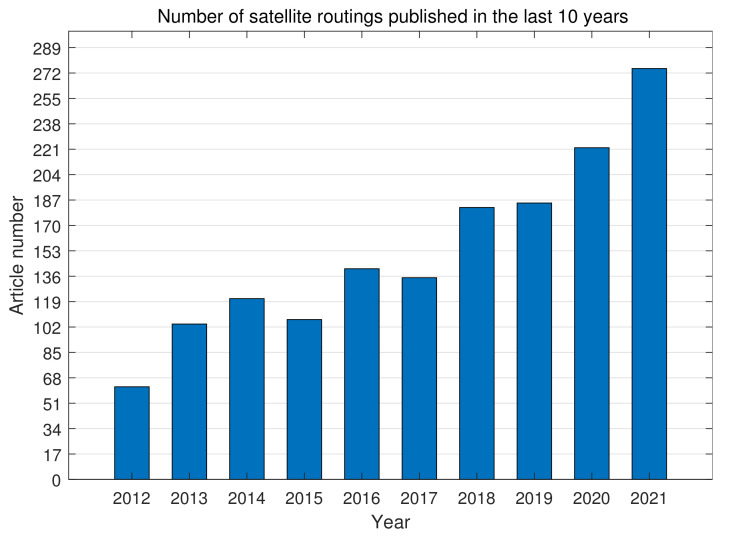
Number of articles.

**Figure 4:**
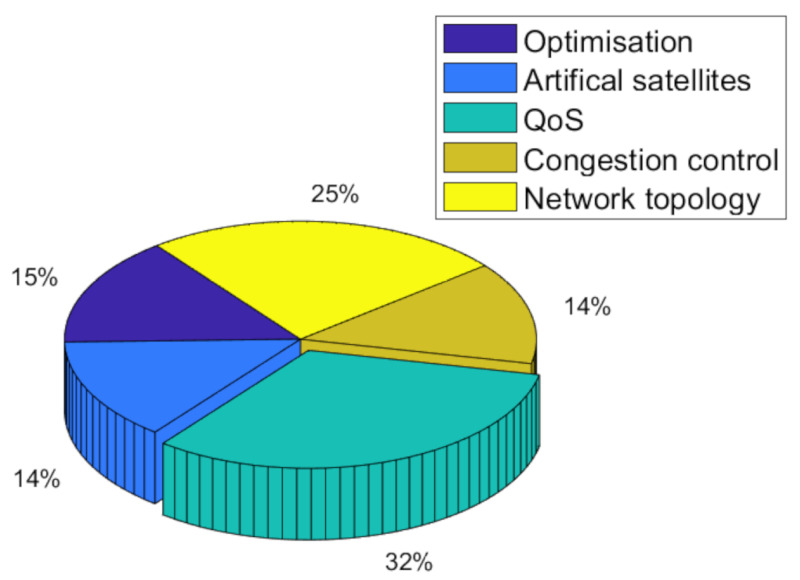
The major studies inside the satellite routings.

Due to the constant movement of satellites and highly dynamic network topology, the inter-satellite routing algorithms have been the focus and challenges of satellite networks. Especially since 2015, the enthusiasm for its research has increased annually. In particular, subjects such as QoS, network topology, congestion control, and intelligent satellites make up the bulk of satellite routing researchers, as shown in Figure 3 and Figure 4 [21].

The dynamical characterization of the satellite network topology has been the focus of current research. As an emerging method, the temporal aggregation graph, is one of the most effective available tools. For satellite networks with limited resources, a resource allocation template was developed in [22]. With the assumption that the topology of each node stays unchanged, the model divides the period of the satellite network into several periods. The network topology for each time interval can be converted into a static topology graph. Given the unique “receive–store–send” mechanism of satellite networks, the scheme adopts the approach of storing arcs to connect the static maps of each timeslot to form a complete network structure. Although temporal aggregation graphs can solve the topology problem of dynamic networks well, it is difficult for the algorithm to adapt to long-term topological changes. As the graph grows, the work in [23] proposed a maximum flow algorithm based on storage time aggregated graph (STAG) to solve the maximum flow problem in disruption-tolerant networks (DTN). STAG utilizes DTN to build the model, incorporating the characteristics of DTN time-variation. In STAG, each node utilizes a bi-directional storage delivery sequence that reflects the correlation between data storage and edge-time gap. STAG can better reflect the time-varying satellite network characteristics, and it aggregates the characteristics of link connections and satellite nodes, resulting in a less complex temporal aggregation graph that takes up less storage resources. In addition, the maximum traffic STAG technology is able to use limited satellite network resources to deliver massive amounts of data services efficiently.

In a given topology, routing is an essential metric determining network performance. The growing size and complexity of satellite networks, however, has made onboard routing more challenging. Various improved routing algorithms have been proposed in academia in response to these problems. Multi-path routing can greatly improve network throughput and end-to-end delay. Using a network-coding-based multi-path routing approach, the complex coordination problem between multiple paths is resolved, thereby improving the data transmission efficiency. In the end, the work in [24] presented a network-coding-based multi-path cooperative routing protocol (NCMCR) to improve the throughput of the LEO satellite networks. The scheme provides a multi-path cooperative routing algorithm that enables dynamic data transmission along multiple paths. The work in [25] employed a time-domain grid model (TNM) to describe the time-varying topology of a large-scale network of small satellite networks (SSNs). The scheme replaces the traditional coordinate positioning method, and satellites can be positioned by the grid. The scheme proposes an efficient grid shortest path routing (NSR) algorithm based on TNM. The work in [26] further investigated the problem of large-scale heterogeneous Internet interference-resisting routing. Firstly, a Stackelberg-based routing strategy for resilience to interference is proposed. Secondly, a deep-reinforcement-learning-based routing algorithm (DRLR) method is used to train it with deep enhancement. Then, a fast response anti-jamming algorithm (FRA) is given to perform anti-jamming decisions. In addition, users can use the DRLR and FRA algorithms to empirically analyze interference decisions and make appropriate anti-interference judgments for dynamic situations under various interference conditions.

Because of the various constraints, compared with the routing algorithms of terrestrial networks, the routing algorithms mainly have the following characteristics: (a) The computing speed and storage resources of the CPU of the on-star facilities are severely limited. (b) In satellite networks, ISL exhibits a mesh feature, making none of the inter-star routing paths unique and possibly generating physical loops. (c) They have a highly dynamic network topology with a higher link-switching frequency, a shorter duration of routes, and an extended transmission time between links. Finally, (d) the services on the star have different QoS requirements and priorities. With the continued maturity of routing technology, scholars have gradually shifted the research focus of routing algorithms to satellite networks. In order to better realize the forwarding of onboard data, the satellite network model should include doubling or multi-layer satellite networks in addition to the typical single-layer satellite networks. Therefore, the academic community divides the existing satellite network dynamic routing into single-layer satellite network dynamic routings and multi-layer satellite network dynamic routings. These will be discussed in more detail in the following sections.

### 4.1. Dynamic Routings in the Single-Layer Satellite Networks

**Figure 5:**
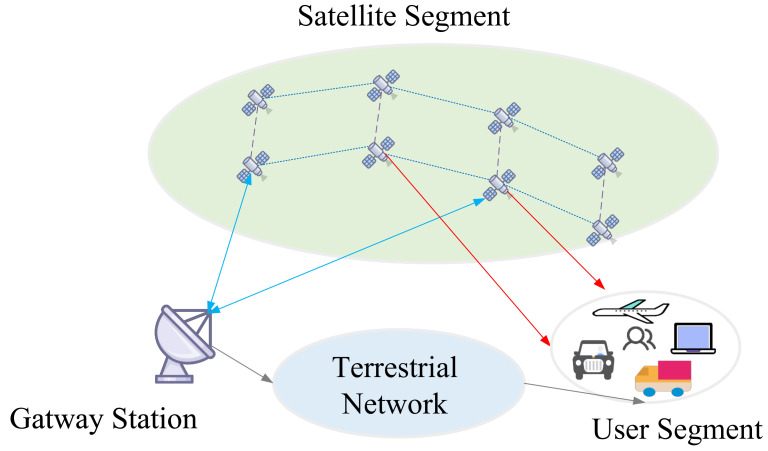
Single-layer satellite networks.

A single-layer satellite network includes satellites with the same orbital altitude and one or more orbital planes. A single-layer satellite network includes satellites with the same orbital altitude and one or more orbital planes. The single-layer dynamic network routings are mainly for the LEO satellite network, whose satellite network structure is shown in Figure 5. Owing to the dynamic LEO satellite topology, this class of routing algorithms is designed to shield it. The three primary methods can be separated into virtual topology, virtual nodes, and topology planning. These are described in the following section.

#### 4.1.1. Topology Planarization

Topology planarization involves treating the entire LEO satellite network as a two-dimensional plane in which satellites do not even change logical positions. Two-dimensional coordinates can represent the satellites to achieve the purpose of shielding the topological dynamics of the network. Table 4 summarizes the works related to the tropological planarized routing algorithm.

The work in [27] firstly used the topology planarization method to shield the highly dynamic nature of the satellite network topology. Based on this, an LEO satellite routing algorithm was proposed. The solution has been experimentally proven to provide low latency, high-frequency rates, and high-quality video delivery to mobile users. However, for such routing protocols, when the satellite network topology changes dynamically based on time, the initially assigned routing paths will no longer be applicable. Thus, in such an LEO satellite network, all satellite links must reacquire new routes, i.e., re-route. However, re-routing mechanisms can cause huge computational overhead in a resource-constrained LEO satellite network, affecting communication quality. Hence, connectionless routing schemes that do not require re-acquisition when the topology changes are more suitable for fast time-varying network topologies. In addition, the route needs further theoretical study in terms of mobility and traffic congestion awareness. As the satellite network technology is improving gradually and the functions of satellite network nodes are enhancing, many satellite nodes can sense geographic location information. The geolocation-based satellite routing algorithm has an inherent advantage over the other satellite routing algorithms. Hence, the research on this type of satellite routing algorithm has fundamental practical significance. The work in [28] developed a new geographic information-based routing algorithm using a delta-type satellite constellation. The algorithm eliminates the need for correspondence between static logical and dynamic networks, significantly reducing the signaling overhead of processing time-varying topologies. To minimize the time latency caused by inter-satellite link switching, satellite nodes in this network are not always required to access the nearest satellite but to remain in contact with one satellite as long as possible. Simulation outcomes show that the scheme can obtain high-efficiency packet routes despite requiring less on-star computing and storage resources. In addition, it does not require the storage of large amounts of network topology data and is therefore scalable. However, the routing algorithm for LEO satellite networks with a particular orbital inclination has certain limitations. In future research, we can consider extending the algorithm to a global coverage network of 3–4 GEO satellites to provide relay datagram services to users in the polar regions. In recent years, DTN has adopted a store-carry and forward transmission strategy to provide route forwarding and storage functions for satellite networks with frequent interruptions without assuming continuous connectivity of network links. However, current dynamic routing algorithms (DRSA), including contact graph routing (GCR), cannot locate end-to-end routes over a series of time-broken links. Therefore, a new extended range routing algorithm (ERRA) was propounded in [29]. The algorithm is a GCR-enhanced routing strategy that can cope with intermittent connections in relay satellite networks. The algorithm involves calculating the satellite orbit information in the ground station and selecting the route. Nevertheless, the algorithm calculates the optimal path on a time-varying topological snapshot and could not guarantee a successful delivery rate and minimal delivery time. In particular, the scheme considers two new metrics of routing tables for link reliability when selecting routing paths, allowing it to make more efficient use of constrained satellite network resources.

To address the issue of LEO satellite networks, the work in [30] devised a geographic IP sub-network partitioning model applicable to the addressing scheme of LEO networks. The segmentation model employs a spherical coordinate system to characterize the location information of LEO satellite orbits and geographic subnetwork regions. On this basis, the author constructed a mathematical model for anomalous addressed users. In the mathematical model, the size and partition of the geographic area will determine the expected proportion of the number of anomalous users. The approach dramatically decreases the number of abnormal users by dividing the ground area into smaller area modules, simultaneously reducing the overhead of partial routing. Nevertheless, while the percentage of anomalous users has diminished during the IP area segmentation, LEO satellite network mobility management and routing table update costs have risen. Thus, the scheme can cut communication expenses by allocating appropriate ground subunits in the routing table updates and mobility management. Satellites can interact with terrestrial users within this LEO network through IP addresses. IP addresses only change when the user or satellite moves to another sub-network, shielding the satellite network from the dynamic nature to a certain extent. However, the specific relationship between partial routing expenditure and abnormally addressed users in the case of increasing abnormally addressed users remains to be studied in depth. However, the model fails to account for network traffic and anomalous user routing optimization. Geographic subnets can reduce the number and generation time of routing tables in LEO networks and maintain routing table stability. An enhanced geographic addressing strategy was propounded in [31]. Motivated by the above, the author developed a framework for the distribution of network user traffic under various geographic sub-networks and optimized the anomaly addressing model. A routing broadcast strategy was presented to address the anomaly addressing optimization issue. The routing solution reduces the system packet loss rate coming from user anomaly bands in the same geographical subnet area. Simulation results indicate that the improved geographic addressing strategy can achieve higher system throughput at a lower routing cost. Simultaneously, the throughput loss caused by anomalous addressing traffic diminishes with the reduction in the geographic sub-network partition area. Nevertheless, the routing broadcast strategy in the present scheme leads to link occupancy. Therefore, we can tackle the problem by designing load-balanced routing algorithms in the subsequent research. In particular, the anomaly addressing strategy introduced by the scheme fails to consider end-to-end delay, route reassembly rate, and other routing cost impact factors, making the performance of the strategy somewhat limited. Packets are routed to a non-specified satellite node in the geographic routing mechanism. Due to the fact that the majority of satellites are not geographically distributed with the earth, it is impossible to accurately divide the surface into the corresponding grid. To address the issue, the work in [32] introduced a new routing path selection algorithm based on MAC addresses. The scheme embeds the location of the terminal in the MAC and separates the topology data of the network from the IP address of the terminal. Based on this, the movement of the terminal may not require a full network-wide update, effectively addressing the routing issue caused by the movement of the satellite. Comparing the above approach to source routing shows that it can guarantee fast and reliable data delivery in diverse traffic scenarios. The algorithm significantly minimizes the end-to-end delay and re-route rate of the network. Meantime, geographic identifiers in MAC addresses support fast routing table lookup and switching. In addition, the routing program also investigates the satellite overload problem and employs a re-routing mechanism. In the following work on satellite routing, we can explore the re-routing mechanism in more depth for different cases.

**Table 4:** The summary of the works related to the topological planarized routing algorithms.

Network Type	Reference	Proposed Algorithm/Scheme	Main Contributions
GEO Satellite network	[29]	A extended range routing algorithm	Considered two new metrics, reliability and routing tables
LEO Satellite network	[27]	A Unicast routing algorithm similar to Mobile IP	Provided low latency and high-quality video delivery
	[28]	A routing algorithm based on geographic information	Reduced network signaling overhead significantly
	[32]	A geographical routing scheme	Reduced end-to-end network latency and reroute rates
	[30]	An addressing scheme based on geographic IP	Reduced the number of abnormal users, decreased routing costs
	[31]	An improved geographic addressing scheme	Reduced system packet loss

#### 4.1.2. Virtual Nodes

In the initial state of the satellite network, the virtual node strategy refers to the projection point of each satellite as a virtual node. Each virtual node has an individual identity. The identity of the virtual node closest to the satellite is used as the identity of the satellite when the satellite is in motion. The identity varies with the satellite motion to shield the satellite network’s topology dynamics. The core idea of such a routing algorithm is to set a fixed logical address for a virtual satellite node. When the satellite moves to the location of the virtual node, its logical address becomes the logical address of this virtual node. As we know, the concept of virtual nodes was first presented in [33]. Numerous researchers have developed their routing schemes based on the approach. Table 5 summarizes the works related to the virtual node-routing algorithms.

The distributed routing algorithm (DRA) based on satellite networks was proposed in [34]. The algorithm takes the polar orbit constellation as the research object, adopts its spatial symmetry to partition the surface space into several specific areas, and sets the corresponding logical spots. Since the polar-orbiting constellation is symmetrical, there will inevitably be a satellite within its range of movement that matches its logical loci, thereby avoiding its high dynamics. However, the method requires satellites with high computing and storage capacity. The routing path generated by such a routing algorithm is not necessarily the optimal route based on the global network topology state information. Multiple shortest paths may exist between two satellites in a satellite network; therefore, determining the optimal shortest path is essential for maximizing network resources. A priority-based adjustable routing (PAR) was proposed in [35]. The approach employs link utilization and historical data to balance the load. PAR takes a distributed routing approach based on the utilization of ISLs and cached data. In addition, the scheme further proposes the enhanced PAR (e-PAR) algorithm to prevent redundant traffic data and better utilize ISLs. The PAR algorithm selects the link with minor output usage. Through an in-depth study of the e-PAR algorithm, the scheme can enhance the stability and performance of the system by adjusting the relevant parameters of the algorithm. The virtual node (VN) approach is used to eliminate the network layer switching problem in fixed LEO satellite systems. However, the physical network is dynamic, and the underlying switching can lead to large packet loss. Therefore, designing a switching algorithm with low packet loss and latency is a crucial issue. Adopting a suitable switching strategy can significantly enhance the performance of a system, particularly in satellite networks with extensive delays. The work in [36] presented a virtual network-based satellite link layer switching algorithm, namely, the virtual node handover operating (VN-HO) algorithm. The solution achieves soft-switching by increasing the number of satellites per orbit. On the improved multi-state virtual network (MSVN) topology, the author suggested a soft-switching algorithm, namely, MSVN-SHO. Experiments have demonstrated that the MSVN-SHO approach has improved performance over the VN-HO in terms of data loss and latency. Therefore, scholars believe that a slight increase in the cost of the system must be taken into account if MSVN-SHO is to be used to its full potential. The work in [37] used a formal model to investigate the dynamics of the LEO satellite network topology and optimized the virtual node model. Compared with the physical topology of the fixed footprint model of satellite networks, the model has the strengths of small snapshot latency and less path variation. Using the small topological snapshot delay to its advantage, the author put forward the dynamic detection routing algorithm (DDRA). The routing solution proposed in this study dramatically enhances the stability and adaptive capability of the network compared to the traditional topological fast photo-routing and distributed routing methods. A VN-based optimal algorithm has a better adaptive capability while hiding the satellite motion characteristics. However, the approach needs to guarantee the system performance when the throughput of the network reaches a high level. Furthermore, in the case of a large number of satellites, employing such a method to find the best path increases the routing calculation overhead of the system.

Current routing algorithms for LEO satellite networks are designed based on path distance. Moreover, the path distance factor only considers the propagation delay and ignores the queuing delay. To solve the concern, the work in [38] introduced a low complexity routing algorithm (LCRA) based on load balancing. Based on the location information of the current node and the destination, the algorithm obtained the optimal path following a distributed routing method. The approach eliminates the need for iterative operations and saves a large amount of on-star computing resources. In addition, each node enables the network to dynamically select the next-hop based on the link state by informing the neighboring nodes of their congestion information. Such schemes achieve lower average queuing delays and packet losses. NS2 tests show that the LCRA method has a better end-to-end delay, throughput, and packet loss than the Dijkstra and DRA methods. In addition, the routing algorithm shows better performance in the LEO satellite network, especially in large-scale user networks. The work in [39] presented an LCPR by exploiting the grid structure of the satellite network topology. In contrast, the conventional algorithm selected the path with the lowest propagation delay based on the latitude and longitude of the current and destination nodes. The LCPR algorithm aims to minimize the computational complexity and the probability of packet loss. The algorithm does not need to store the routing table in the satellite, which effectively decreases the space complexity. The whole algorithm has no iterative process, which makes the time complexity reduced to a certain extent. In addition, the algorithm selects the next-hop adaptively according to the probability distribution, which contributes to the balancing of the service load. Compared with Dijkstra’s shortest path (DSP) and DRA algorithms, LCPR exhibits superior performance in terms of combined throughput and packet loss rate. To ensure that packets can reach their destinations efficiently even in the presence of frequent link and satellite node failures, the work in [40] suggested a local repair-based, disruption-resistant, on-demand routing protocol (DODR) for LEO satellite networks. DODR can be considered as an improved ad hoc on-demand distance vector (AODV) routing algorithm [41]. The main objective of DODR is to minimize end-to-end delay and efficiently transmit packets in case of link or node failure. The employed algorithm routes reply packets in the path discovery and a local repair strategy to quickly repair broken paths with low overhead, improving the real-time network performance. Compared to location-assisted on-demand routing (LAOR) [42], snapshot routing protocols, and source retransmission routing in case of link failure, DODR maintains low packet loss and end-to-end delay performance even in link failure [43].

#### 4.1.3. Virtual Topology

With the virtual topology strategy, the operational period of the satellite is divided into n time intervals. The satellite network topology remains unchanged between time gaps, so routing optimization can be performed using terrestrial network protocols. The virtual-topology-based routing algorithm only needs to calculate the route at the current time. This approach uses visible satellites to build a visible matrix within each time slice and calculates the optimal route based on traffic demand. The ultimate goal is to fully utilize the limited satellite link resources. However, a link allocation algorithm generally adopts a centralized calculation approach and cannot sense network congestion. Table 6 summarizes the works related to the virtual topology routing algorithms.

Dynamic virtual topology routing (DV-DVTR) was introduced in [44]. First, a virtual-topology-based connection-oriented routing algorithm based on ATM was proposed. DV-DVTR is a system period of the satellite network divided into time slices. In each time slice, the satellite network topology is considered to be fixed. The V-DVTR algorithm can be categorized into two phases, the discrete-time virtual topology setup (DTVTS) and the discrete-time path sequence selection (DTPSS). In the DT-VTS, the DT-DVTR algorithm dissociates the LEO satellite network topology into a set of static topologies. In the DTPSS, the classical Dijkstra method is adopted to perform the routing operations. The optimized route is uploaded to each satellite and is corrected at each moment. The DV-DVTR discretizes the topology of a satellite network into a series of static topologies for the first time. However, such an algorithm cannot solve the rerouting problem caused by link switching. The Finite State Automata (FSA) routing algorithm was presented in [45]. The algorithm considers the packet link, satellite network topology, and network traffic to maximize the utilization of the ISL. Like the DV-DVTR, the FSA segments the satellite network topology into a finite number of time slices, and the algorithm is a connection-oriented routing algorithm. Nevertheless, the difference is that FSA treats the topology of each moment block as a "state" and models it as a finite state model. The scheme utilizes an iterative approach to find the best link and route assignment for each node. The strength of FSA is that it can maximize the use of the network’s resources. However, the algorithm also has some limitations. First, the shortest route is not a selection criterion for routes. Secondly, implementing the dynamic link assignment method in LEO satellite networks is difficult. In conclusion, the method is operationally complex. The first study of the multi-path routing problem for satellite networks is compact explicit multi-path routing (CEMR) [46]. The basic idea of CEMR was to utilize the Path-ID path identifier to encode the path. Based on that, a new satellite network model based on a dynamic virtual topology was developed. In the model, when the time interval is small enough, the cost of each satellite link is considered constant over that time interval. The multi-path routing mechanism of the CEMR mainly includes route discovery, route maintenance, and traffic distribution. In the algorithm, the sum of propagation delay and queuing delay constitutes the time delay. In addition, the idea of global path planning of the CEMR algorithm is utilized to achieve explicit multi-path routing for LEO satellite networks. Meanwhile, the CEMR algorithm can reduce the LEO satellite network delay, improve the system throughput, and balance the load since the routing algorithm and grouping policy are not covered. Grouping policies without improvements may generate too many snapshots, and routing algorithms cannot be applied in this case. For that, the work in [47] modified the ant algorithm and combined it with routing methods. The improvement in the ant colony algorithm generally includes two different approaches to an ant system, with an elitist strategy as the elite approach and the rank-based version of the ant system as the ranked approach. However, the rank-based method takes too long to run and fails to adapt to the rapid topology changes in the satellite network. Therefore, this scheme uses ant systems with an elitist strategy as elite to improve the ant colony algorithm. The improved ant colony algorithm shortens the convergence time and reduces the generation of snapshots. In addition, the solution updates only the pheromone concentration on the optimal route to prevent the algorithm from falling into local optimality. On this basis, the global optimal routing path can be obtained by reasonable adjustment of each parameter. The experiments prove that the routing algorithm combined with the grouping strategy has a better performance.

However, the VN-based routing scheme is incapable of enabling multiple satellites to serve a single coverage area of the network simultaneously. In [48], a multi-state virtual network (MSVN) topology was designed to address the issue. The topology allowed multiple satellites to cover a single area simultaneously. In addition, the scheme investigated the potential switching mechanisms in fixed satellite systems. Meanwhile, the soft handover algorithm for MSVN-based satellite networks (MSVN-SHO), the VN-HO, and the MSVN-SSHO were proposed. Despite the increased cost, the switching algorithm based on the MSVN system has the benefit of being faster and smoother compared to the VN-HO switching algorithm. The work in [49] presented a routing algorithm based on virtual topology snapshots, namely, the DDRA. The DDRA guarantees the benefits of the topology snapshot algorithm while offering small transmission latency and high throughput rates. In addition, the DDRA can adapt well to network changes and avoid communication delays caused by queuing and link failure. Experiments prove that the DDRA outperforms the traditional topological fast routing and distribution path selection methods. Among the currently available routing algorithms for satellite networks, little research has been conducted on the trade-off between the survivability of routes and the computational overhead of routes. For this reason, a new routing protocol with traffic prediction, namely, the distributed traffic balancing routing protocol (DTBR), was proposed in [50]. DTBR not only maintains high routing capacity under lesser load conditions but relieves congestion in the network and achieves traffic balancing through the cost factor of ISL. In addition, DTBR does not add any additional communication overhead, except for satellites with a large number of failures in the network.

The work in [51] integrated the deep-first-search (DFS) and the Dijkstra algorithm for SDN-based massive LEO mobile satellite networks. DFS is a typical path selection approach. However, the performance of DFS depends on the number of nodes in the network. The Dijkstra method decreases the computational overhead by first finding the shortest path between the destination and source nodes during the solution process. Therefore, the scheme takes full advantage of both the DFS and Dijkstra algorithms to improve routing performance. Simulation results show that the joint DFS and Dijkstra algorithms outperform the traditional DFS algorithm. However, the algorithm sacrifices metrics such as end-to-end delay to some extent. For this purpose, a new distributed routing hybrid model was designed in [52]. The scheme utilizes mobile agents (MAs) to collect routing state information. The simulated results show that the model outperforms other routing models in terms of average delay, packet loss rate, and queuing delay. The explosive growth of communication traffic and the uneven distribution of users in LEO satellite networks have led to the emergence of link congestion control protocols. Therefore, the work in [53] suggested a distributed congestion control routing protocol (DCCR) for LEO satellite networks based on flow classification. The solution meets the computing power required by LEO satellite networks and effectively reduces the computing overhead of centralized routing. DCCR classified services by latency and throughput requirements. A distributed routing scheme was applied to route the different services to reduce the load in the network while at the same time ensuring the delay and throughput requirements of the services. Simulation results show that DCCR can better accommodate QoS requirements, reduce queuing time in the network, and maintain low-latency in high-load conditions. Due to the limited resources of the satellite network, the work in [54] designed a virtual network structure that can be updated in real-time to match the topology of the network. The method adopts a virtual topology that allows pre-storing the required topology and routing tables, thus saving a large amount of satellite computing resources with reduced system latency. Since the satellite network is cyclical, the topological changes of the satellite network are also cyclical. In each cycle, we can analyze and calculate the topology of the satellite in advance. The calculated network topology can find the optimal path between any two nodes in the network, reducing the communication overhead of the network, and the routing algorithm is implemented. Through OPNET software modeling and simulation, the algorithm can obtain the average delay between network nodes, network throughput, and other related metrics under various bit rate conditions. Efficient and secure forwarding of packets in the Satellite Internet of Things (S-IoT) is challenging due to the dynamic changes in satellite network topology and node states. Therefore, the work in [55] proposed an S-IoT adaptive routing algorithm based on improved dual-Q learning. The scheme applies reinforcement learning to S-IoT routing policies, enabling them to adapt to dynamic changes in the S-IoT topology and node states. First, the approach sees the whole S-IoT as a reinforcement learning system, treating the satellite and ground nodes as intelligence. Each node needs to maintain two Q-tables for forwarding and evaluation in S-IoT. Second, for the optimal Q value, the proposal utilizes the network congestion level and node state to improve the hybrid Q value, reward value, and discount factor, respectively. The algorithm can achieve more efficient and secure routing in highly dynamic environments than other routing algorithms. Future studies can replace two Q-tables with two neural networks due to the increase in S-IoT nodes. However, double-Q learning has been rarely used in satellite routings. In order to improve the accuracy of routing optimization, more in-depth research on this type of learning algorithm is needed in academia. Comparisons of the key performance of partial dynamic routings in single-layer satellite networks are shown in Table 7.

**Table 6:** The summary of the works related to the virtual topology routing algorithms.

Proposed Algorithm/Scheme	Reference	Features/Advantages	Contributions
A discrete-time routing scheme	[44]	Divided into discrete-time virtual topology setup and discrete-time path sequence selection	Discrete the topology of the satellite network into a series of static topologies.
A new routing algorithm based connection-oriented	[45]	Considered the topology of each moment as “state”, maximized the resources of the network	Found the optimal path for each node in each case
A explicit multi-path routing	[46]	Mainly included route discovery, route maintenance, and traffic distribution	Effectively reduced latency in the LEO satellite network, improving system throughput and load balancing
An improved ant colony algorithm	[47]	Updated only the pheromone concentration on the optimal route to avoid the algorithm from falling into a local optimum	Shortened algorithm convergence time and reduced snapshot generation
Soft handover algorithm for MSVN-based satellite networks, virtual node handover	[48]	Faster and smoother	The topology allowed multiple satellites to cover a single area at the same time
A dynamic detection routing scheme	[49]	Smaller transmission latency and higher throughput rates	Adapted well to sudden changes in the network, avoided communication delays caused by queuing and large packet loss due to link failure
A traffic balanced routing scheme	[50]	Sustained high routing capacity under low load conditions, relieved network congestion at the cost of ISL	No additional communication overhead is added, achieved traffic balancing
A routing algorithm combining deep-first-search and Dijkstra algorithm	[51]	Utilized the strengths of both DFS and Dijkstra algorithms	Reduced computing overhead, improved routing performance
A new distributed routing algorithm	[52]	Utilized the advantages of both DFS algorithm and Dijkstra algorithm	Outperformed other routing algorithms in different metrics such as average delay, packet loss rate, and queuing delay
A distributed congestion control routing algorithm based on flow classification	[53]	Effectively reduced computational overhead of centralized routing, alignment of different services based on distributed routing schemes	Adapted to QoS requirements and reduced queuing time in the network
S-IoT adaptive routing scheme	[55]	Improved hybrid Q value, reward value, and discount factor using the network congestion level and node state, respectively	Enabled more efficient and secure routing in highly dynamic environments

### 4.2. Dynamic Routings in the Multi-Layer Satellite Networks

**Figure 6:**
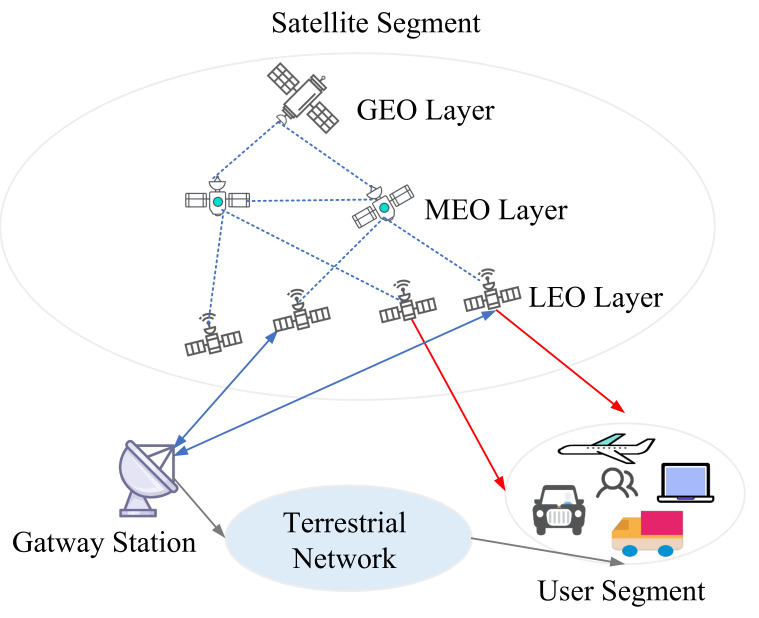
Multi-layer satellite networks.

Multi-layer satellite networks were created to overcome the single-performance layer’s limitations. As shown in Figure 6, a multi-layer satellite network includes satellites with different orbital altitudes and has a more complex network structure, such as LEO/MEO, LEO/GEO, MEO/IGSO, LEO/MEO/GEO, etc. [56]. The dynamic routing in multi-layer satellite networks is designed with a great deal of freedom due to the diversity of network architectures. The satellites are grouped in different layers according to their distance from the ground control center. Short distances are routed using only LEO satellites, while long distances are routed via MEO satellites or GEO satellites. In addition to grouping, due to the hierarchical structure among different satellite nodes, dynamic routing strategies can be considered by dividing labor among individual satellites, such as dynamically adjusting routes based on delay or link congestion. When the number of routing hops for the LEO layer satellite exceeds a specific delay threshold or when the current link is congested, the system activates the inter-satellite link between the GEO satellite and the LEO satellite before incorporating the GEO satellite into the route. Based on academic and industry dynamic routings for multi-layered satellite networks, they can be classified as SDN-based, QoS-based, and traffic-balancing dynamic routing.

#### 4.2.1. SDN-Based Dynamic Routing

**Figure 7:**
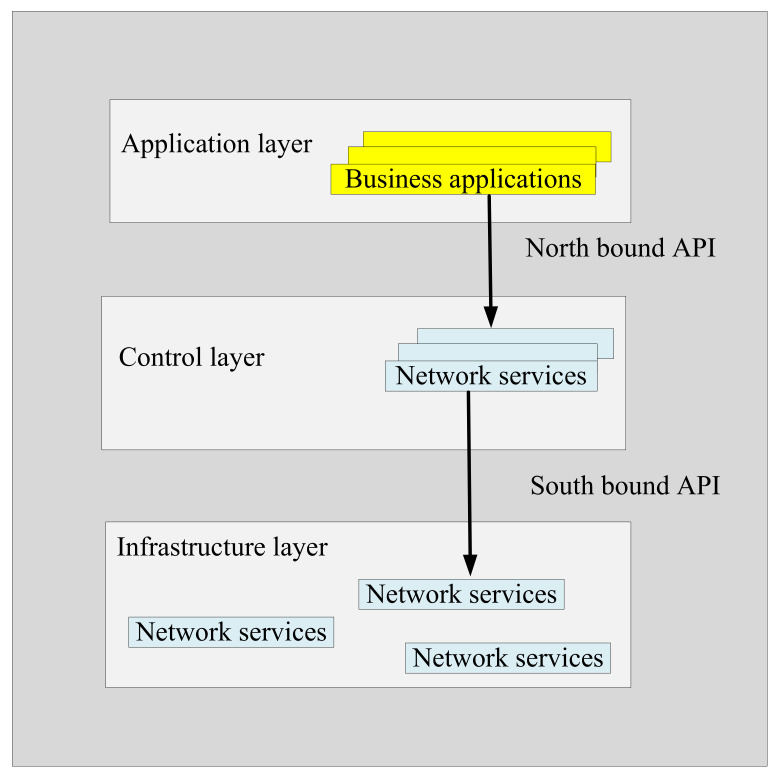
Network vision with SDN.

**SDN-Based Network:** SDN is an emerging network architecture that separates network control operations from network forwarding services and provides programmability of the controls [57]. SDN dramatically increases the network architecture’s flexibility, effectively enabling intelligent routing algorithms based on machine learning to control network traffic allocation, QoS requirements, etc., as shown in Figure 7.

**Figure 8:**
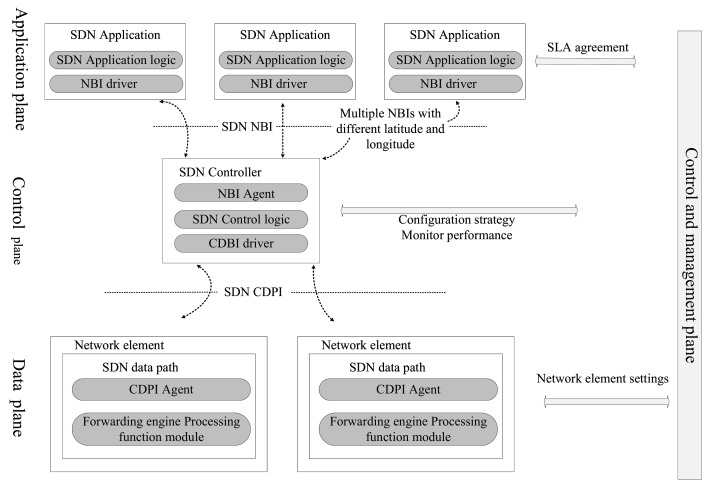
SDN Architecture.

SDN is a new network architecture that separates the data plane from the control plane and supports network programmability. It can realize the centralized control of data and optimization of network resources. The above definition of SDN is the consensus in the industry, but the definition of SDN varies among different standardization organizations. Figure 8 shows the reference architecture of the SDN definition introduced by the ONF organization. The SDN architecture defined by ONF is divided into four parts: the data plane, the control plane, the application plane, and the management plane. Each part communicates through different interfaces.

**Figure 9:**
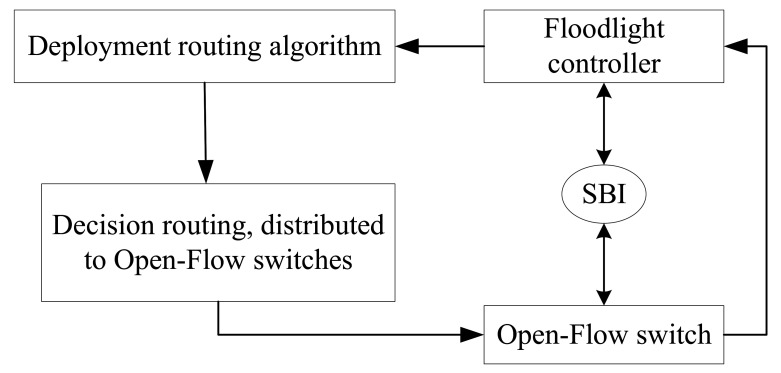
Routing process of SDN.

**Routing mechanisms for SDN:** The data-flow-based routing and forwarding strategy is adopted in the SDN network architecture to realize the effective coupling between the controller and the switch. Before the data flow reached the Openflow switch, the SDN controller sent the policy to the underlying switch. When the passive mode waited for the data flow to reach the Openflow switch, the underlying switch asked the upper SDN controller how to handle the exit. When the controller decided, it sent a flow table to all switches. Figure 9 shows the basic steps of SDN Grid routing.

**SDN-based dynamic routings in Satellite Networks:** The SDN architecture defined by ONF is divided into four parts: the data plane, the control plane, the application plane, and the management plane. Each part communicates through different interfaces. The software-defined satellite networks (SDSN) were proposed in [58]. In SDSN, GEO satellites act as the control plane, MEO and LEO satellites act as the data plane, and ground stations act as the management plane. Among them, the control plane is responsible for distributing commands from the management plane to the data plane, monitoring the real-time status of the satellite network, and then feeding it back to the management plane. However, the highly dynamic topology of multi-layer satellite networks poses new challenges to the existing SDN control architecture. Among SDSNs, snapshot routing is the most basic and representative. Table 8 summarizes the works related to the SDN-based dynamic routing algorithms.

In 2017, the work in [59] developed SDSN snapshot routing and validated it with a typical Iridium architecture as an example. The approach utilizes the satellite link reports generated by STK to divide the entire Iridium architecture into 44 snapshots. On this basis, the Dijkstra algorithm is used to solve for each snapshot route and transform it into a static routing table. The system sends this static routing table to each satellite before performing the snapshot transformation. The new routing table is used to process the packets when the snapshot transformation is performed. Through the test, the routing tables of all satellites were able to be updated correctly, ensuring proper communication between the satellites. The work in [60] introduced the idea of decoupling the control plane and data plane into the satellite network and designed a controller model. The model decreased the communication cost of the network and increased the convergence speed of routing. A routing strategy based on theAmoeba algorithm and Amoeboid–Ripple Spreading routing algorithm was designed. The experimental results show that the algorithm is able to perform the network routing task with a low packet loss rate and a small number of routing hops. In multi-layer satellite networks, a large number of satellites and the dynamic topology make the multi-path transmission control protocol (MPTCP) the preferred routing protocol. To address the existing issues of the MPTCP, in 2018, the work in [61] proposed an SDN-based MPTCP routing algorithm. The solution determines new satellite routes by using each link’s remaining bandwidth, solving the “bottleneck” problem, and adapting to changing network loads. In addition, by adjusting the shared satellite links, the throughput of the network is further enhanced. To achieve more flexible traffic scheduling and QoS guarantees in multi-layer satellite networks, the work in [62] proposed an integrated framework for satellite-ground satellite communication networks (SERvICE). On this basis, two heuristic algorithms, the QoS-oriented satellite routing (QSR) algorithm and the QoS-oriented bandwidth allocation (QBA) algorithm, are designed to ensure the QoS requirements of multiple users. Inspired by the network framework, in 2019, the work in [63] presented a three-layer SDN-based satellite network model. The GEO satellite was the control satellite tasked with calculating the optimal communication link and resource allocation. Meanwhile, to improve the self-adaptive capability of the network, the GEO satellite can adjust the inter-satellite links in real time based on the network status. In addition, the scheme investigates an adaptive routing algorithm (ARA) based on the model. The algorithm finds the shortest communication path between satellites and optimizes the routing in real-time. The work in [64] combined machine learning and an SDN to solve dynamic network topology and link traffic awareness in multi-layer satellite networks. The scheme adopts the deep deterministic policy gradient (DDPG) algorithm for routing optimization. The algorithm can make appropriate routing decisions based on the real-time link state. In particular, utilizing the long short-term memory (LSTM) neural network, the algorithm improves the sensing capability between the associated satellite links.

With the network controller control center (NOCC), the SDSN provides flexible configuration and scheduling for satellite nodes within the network. This significantly lessens the complexity of the satellite network architecture, onboard storage, and computational power requirements, especially for LEO and MEO onboard storage and computing. The SDSN is a new type of hybrid control network. Due to the excessive distance between the inter-satellite links, dynamic routing is challenging to adapt to the requirements of the network, especially for delay-sensitive services. Therefore, in SDSN, using the snapshot routing algorithm is still the most dominant path choice. However, the traditional snapshot routing method causes the GEO satellites to become more computationally intensive and results in the loss of data. In addition, it is inconceivable to accurately describe the topology of inter-satellite links due to the traditional snapshot routing. To ensure minimal computing power and storage pressure on GEO satellites in SDN systems, optimizing routing performance in dynamic topologies has become an urgent need for large-scale multi-layer satellite networks. In 2020, the work in [65] designed an adaptive snapshot routing strategy (ASRS) to optimize snapshot routing performance. ASRS is divided into two main categories. One is for the snapshot segmentation of multiple domains, and the other is the snapshot routing for various services. First, a multi-domain snapshot partitioning method is used to obtain each region’s set of snapshot sequences. Then, the snapshot routing algorithm is employed. Satellite links select and combine snapshots based on service arrival and transmission times. ASRSs can effectively reduce the bandwidth resource loss of the channel. Simultaneously, the efficiency of snapshot routing has been improved. The simulation compares ASRS with the traditional snapshot routing algorithm in Open Network Operating System (ONOS). The results show that the ASRS algorithm has significant superiority in satellite network resource utilization and QoS. In multi-layer satellite networks, saving energy becomes a critical issue due to the limitations in network resources. Since the SDSN has integrated control over the entire network resources, it dramatically improves the utilization of satellite resources [66,67]. In a sense, SDSN is a “green” satellite network. However, many energy losses in SDSN cannot be ignored. Therefore, reducing the non-negligible energy consumption in the network can contribute to the energy saving of SDSN. A new energy consumption model for multi-layer satellite networks was designed in [68]. For the model, an SDSN topology generation algorithm was introduced. The algorithm considers the link switching energy consumption, as well as the inter-satellite link energy consumption. Second, a DDoS control scheme was given for the vast energy consumption caused by abnormal traffic in the network. In SDSN, the abnormal traffic generated by DDoS attacks consumes many of the network’s resources and severely disrupts the storage and forwarding of normal traffic in the network. As a result, the overall resource utilization of the satellite network decreases while the overall energy consumption increases. Therefore, to ensure the security of the satellite network and its efficient resource use, an intelligent traffic mitigation strategy is needed. Through performance evaluation, the new network topology generation algorithms and DDoS attack mitigation methods effectively reduce network energy consumption.

In multi-layer satellite networks, single-path routing can no longer accommodate the QoS requirements of various services. In single-path routing, all packets must select the optimal path of the current link for transmission, wasting the network’s limited spatial resources. In addition, due to the solid dynamic nature of multi-layer satellite networks, the switching of single routing paths also takes time, resulting in a certain degree of network delay. On the contrary, multi-path routing can provide path choices based on QoS requirements, improving network QoS. In 2021, utilizing the global topology information of SDNs, the work in [69] designed a multipath routing algorithm (MPRA) based on the ant colony algorithm. In order to minimize the average length of the links while minimizing the number of inter-layer link switches, the method employs the optimal minimum switching strategy (OMHS). The improved ant colony algorithm reduces path duplication and selects high-quality routing paths that meet QoS requirements. The algorithm uses different ant colonies to optimize multiple QoS-compliant routing paths. In multi-layer satellite networks, multipath routing provides more options for data transmission and improves network security. However, due to the limitations of the heuristic algorithm, the multi-path routing algorithm fails to guarantee that all found routes are optimal and meet the QoS requirements. Therefore, in the following research, using joint machine learning to train the algorithm can reach the expected goal and generate optimized routes. At the same time, the algorithm is stable and resistant to destruction. The work in [70] designed a LEO–GEO satellite network model based on the SDN architecture. The model includes three GEO satellite controllers and one ground controller. For two-way session services, the solution sets a high-priority queue to ensure its priority. Moreover, the approach optimized the LCRA proposed in [38]. Since there are no iterations in the computation process, the on-star storage cost is greatly reduced. QoS-aware routing (QoSRA) outperforms other routing methods in average delay, packet loss rate, and network throughput. To better verify the feasibility of various routing algorithms and the reconstruction of inter-satellite neighboring links, an experimental platform for SDN-based multi-layer satellite networks was designed. An ONOS-based SDN controller is used in the testbed to control multi-layer satellite networks. In addition, the demo platform is set up with 200 virtual satellite nodes to verify routing reconstruction. The integrated satellite–terrestrial network (ISN) is an emerging network framework to complement terrestrial 6G networks. In 2022, the work in [71] combined SDNs, artificial intelligence techniques, and fuzzy logic to design a new fuzzy CNN-based multi-task routing (FCMR) algorithm. The solution utilizes a two-stage LEO–GEO satellite network for long-distance, high-throughput, and low-latency data transmission. Based on the dynamic characteristics of the satellite, the scheme employs the GEO satellite and the ground computing center (GCC) as a common control plane. The GEO satellite mainly completes the collection of load data for each period, forming a multidimensional matrix. The GCC captures historical traffic data from the GEO controller for training and updating the convolutional neural network (CNN) model. The GEO satellite applies the trained CNN model for path planning and transmits the stream data stream to the LEO satellite. In addition, considering that CNN judgments may contradict the quality of experience (QoE), FCMR uses fuzzy reasoning to evaluate the task requirements, improve the output efficiency of CNN, and achieve the best ISN path assignment. To ensure QoE and load balancing, FCMR applies optimal ISLs to schedule multiple tasks. Simulation results show that FCMR has better network throughput, path-finding efficiency, and ISN congestion control. Compared with the basic CNN and Dijkstra routing algorithms, the proposed FCMR can employ fuzzy logic to enhance the decision flexibility of CNN. However, this subsection describes the latest dynamic routing algorithms for SDN-based multi-layer satellite networks. Moreover, the routing optimization algorithms in this subsection apply only to one satellite network model and no other constellation model in the longer term. Therefore, in future research, we need to further investigate and explore the suitable routing algorithms in all constellation models.

**Table 8:** The summary of the works related to the SDN-based dynamic routing algorithms.

Network Structure	Reference	Proposed Algorithm/Scheme	Results
SDN-MPTCP	[61]	A MPTCP routing scheme	Be adaptable to changing network loads, further increased throughput of the network
SDSN	[68]	An algorithm for topology generation	Reduced network energy consumption effectively
GEO–MEO–LEO	[60]	A routing strategy based on amoeba algorithm and amoeboid–ripple spreading routing algorithm	Reduced communication costs of the network, improved convergence speed of routing
	[64]	An adaptive routing algorithm	Found the shortest communication path between satellites, optimized routing in real-time based on the state of the satellite
	[65]	A snapshot routing scheme	Reduced bandwidth resource loss in the channel effectively, improved efficiency in the use of the satellite network resources
SERvICE	[62]	QoS-oriented satellite routing algorithm and QoS-oriented bandwidth allocation algorithm	Guaranteed QoS requirements for multiple users
GEO–LEO	[70]	A QoS-aware routing scheme	Reduced onboard storage costs dramatically, capable of meeting various QoS requirements better
	[71]	A new fuzzy CNN-based multi-task routing algorithm	Improved output efficiency of CNN, achieved optimal ISN path assignment, ensured QoE and load balancing
SDN-SGIN	[64]	An optimization algorithm for routing based on machine learning	Updated routing based on real-time link status, improved correlated satellite link sensing capability
MEO–LEO	[69]	A multi-path routing algorithm	Reduced duplication of routes, calculated multiple QoS-compliant routing paths simultaneously, improved network security

#### 4.2.2. QoS-Based Dynamic Routings

Satellite networks are developing towards merging with the terrestrial networks. Therefore, more services will flood into the satellite network, especially for multi-layer satellite networks. Effective QoS dynamic routing algorithms must be designed to meet the QoS needs of multi-layer satellite networks. Meeting QoS requirements is a multi-parameter optimization problem, considered polynomial-complete. Several QoS-based dynamic satellite routing schemes have been proposed one after another. Table 9 summarizes the works related to the QoS-based dynamic routings.

Based on the time slot division approach, the work in [72] proposed a new dynamical hierarchical distributed QoS routing protocol (HDRP). The algorithm utilizes QoS metric information, including delay and bandwidth, to calculate the routing table while ensuring real-time multi-media service transmission. However, the satellite group management scheme fails to guarantee the reliability of satellite data transmission. For resource-constrained, multi-layer satellite networks, finding low-cost routes that meet the QoS requirements of all users is a significant challenge. Based on the idea of ranking optimization, the work in [73] designed a new algorithm for multi-QoS path selection. Compared with the existing SPF protocol, the approach can effectively balance the network traffic load and prevent packet loss. Since the algorithm is ground-based, it avoids onboard network constraints. The experimental results show that the algorithm can effectively perform routing under all pre-defined QoS parameters and shows better performance in hierarchical satellite networks. With the application of heuristic algorithms, the path selection capability of dynamic routing for QoS has been greatly improved. By improving the virtual topology policy, the work in [74] introduced heuristic algorithms to meet the QoS requirements of MLSNs. The method uses ant colony, forbidden search, and genetic algorithms to optimize multi-layer satellite network routing. Simulation results show that the algorithm obtains more QoS guarantees in packet loss rate, link congestion, and other performance aspects compared to the shortest path optimization algorithm. The authors of [75] designed a novel GEO–highly elliptical orbit (HEO)–LEO satellite network architecture to improve global coverage. The QoS-aware multi-point transport routing (QAMRP) scheme was proposed based on the network architecture. In QAMRP, the multi-cast tree is built using LCT, reducing tree overhead and meeting QoS requirements. Compared with the shortest path tree (SPT) strategy algorithm, the algorithm is superior in tree delay, tree overhead, and multi-point transmission failure. Each satellite is a topological node in multi-layer satellite networks, so the whole network is a graph matrix. However, each satellite can only acquire QoS information of neighboring ISLs. Therefore, based on link congestion and QoS awareness, the work in [76] adopted a new distributed routing algorithm to estimate the global QoS conditions among satellite nodes. Simulation experiments show that the method has excellent network control and management capabilities. However, this scheme fails to address the root of the QoS routing issue. Therefore, the easiest way to meet QoS requirements in multi-layer satellite networks is to develop a routing scheme.

In 2016, utilizing the ant colony optimization algorithm, the work in [77] designed a new adaptive QoS dynamic routing. The algorithm determines the optimal QoS path for the destination node by collecting the QoS status between the source and destination satellite nodes. This method effectively prevents overloading in the satellite nodes during path selection. In addition, the proposed algorithm also optimizes QoS parameters based on specific satellite scenarios, improving convergence. Compared to the traditional distributed QoS routing based on ant algorithm (DQA), the scheme has a better end-to-end delay and QoS state values. However, the algorithm ignores QoS relationships, affecting network use. The work in [78] proposed a multi-service routing algorithm for GEO–LEO dual-layer satellite networks. The scheme uses the multi-layer satellite network’s dynamic topology to provide broadband service. Correlation weights can better regulate latency services by considering the remaining bandwidth and latency. Simulations show that the proposed method reduces the latency of high-priority services and improves the QoS of low-priority services. In addition, the throughput capacity of the proposed method has significant advantages in the case of high service volume. Meanwhile, it effectively solves the problem of multi-service routing and improves the communication quality of the multi-layer satellite networks. However, satellite networks are characterized by intermittent connections, extensive delays, and time-varying topologies, limiting link QoS requirements. In 2017, the work in [79] introduced a QoS algorithm for IP satellite networks. By taking link throughput and end-to-end delay as path cost factors, the algorithm solves for a demand satellite route that meets link QoS. Simulation analysis identified the most critical causes of link delay: queue delay and global link throughput. However, congested satellite links cause longer delays for high-throughput traffic. When there is a blockage in the LEO satellite network, the algorithm selects the routing path in the GEO satellite network. However, the current approach focuses largely on link latency and ignores other factors. Hence, the work in [80] designed a new QoS-enabled routing strategy. The scheme utilizes a temporal aggregation graph (STAG) to implement QoS-based routing. QoS dynamic routing is a joint optimization problem of minimizing transmission delay and maximizing throughput based on service requirements and network latency. The algorithm addresses the shortest path problem and the multiple optimal traffic paths for a given transmission delay. In addition, the algorithm maximizes traffic and minimizes latency while reducing complexity. Finally, the effectiveness of the algorithm is verified by theoretical analysis. However, the model fails to consider the requirements of task transmission and cannot give a specific QoS routing scheme. Therefore, in 2019, the work in [81] engineered a QoS multiple routing (QSMR) algorithm. The algorithm focuses on the QoS issue for multiple transmission tasks on multi-layer satellite networks. The proposed algorithm considers traffic and delay requirements based on time-varying network resources. However, the algorithm leaves out the effect of the satellite transceiver on the routing performance. Therefore, future research can focus on QoS routing with limited transceiver resources.

In multi-layer satellite networks, the rapid growth in the type and volume of services has made the QoS requirements of users even more demanding. Traditional QoS routing algorithms consider only a single QoS demand, ignoring their priority relationship, which affects network resource utilization. In 2018, the work in [82] exploited the feature vector method to set weights for varied QoS state values. The link evaluation index is composed of the priority function and the evaluation criterion function, and the optimal path that meets the QoS requirements is solved. The algorithm uses a GEO satellite as the link information controller and an LEO satellite to update the link information in orbit. The simulation results show that the algorithm effectively solves the QoS guarantee issue of multi-media resources under a large load. In addition, the algorithm can distinguish different QoS requirements and balance network traffic, further improving network resource utilization. However, the algorithm fails to guarantee the QoS of data transmission. Based on this, making use of the ant colony algorithm, the work in [83] designed a dynamic QoS routing scheme (SRADR). The method improves and optimizes the heuristic approach to meet the communication requirements of multi-layer satellite networks. SRADR sends N ants from the source node to the destination node to find the best QoS path. The algorithm primarily consists of two processes: dynamic route lookup and dynamic routing table updates. Based on the optimization objective function, dynamic route finding is used to seek out the best path to different destination nodes. In multi-layer satellite networks with high dynamics and heavy performance jitter, SRADR requires setting a dynamic route detection cycle. During the period, a number of ants can be used to discover the optimal path to different destination nodes, and complete the dynamic update of the routing table. Based on the ant colony algorithm, in 2019, the work in [84] designed a QoS routing algorithm using the ant colony algorithm. The average delay of the algorithm is measured in seconds, the latency jitter of each path is measured in milliseconds, and the packet loss rate of each path is only 0.11%, which guarantees the QoS requirements of multi-layer satellite networks. However, the algorithm guarantees the QoS requirements by sacrificing the satellite network communication overhead. Meanwhile, the authors proposed a multi-constrained QoS routing algorithm for multi-layer satellite networks. Utilizing the QoS information in the current link as an essential basis for ants to select the next-hop node, the algorithm enhances the heuristic function of the ant colony algorithm. In the LEO–MEO–GEO satellite network, the time division routing protocol (TDRP) is applied to classify the satellites in various layers [85]. Each layer of satellites can be treated independently, saving computing time. In particular, the GEO satellites are responsible for the routing table calculation and are entirely independent. In addition, after calculating the routing table for their respective layers, MEO and LEO satellites must send it to their group members, making them relatively independent. Since multi-layer satellite networks have limited resources, to make more appropriate use of the constrained network resources, QoS satellite routing is provided for various types of services. To compare the existing QoS routing algorithms, various types of services have different QoS requirements. If all services are processed under the same QoS conditions, the QoS requirements of the higher-priority services will be decreased. Therefore, based on priority and failure probability, the work in [86] proposed a routing algorithm for LEO–MEO satellite networks. To a certain extent, the approach can reduce the packet loss rate and service delay, improve the network throughput, and guarantee the QoS requirements of the service. Nevertheless, to ensure the QoS for high-priority services, low-priority services are flooded onto MEO satellites, causing traffic congestion. Furthermore, the algorithm considers only two QoS parameters: bandwidth and latency. In the next QoS dynamic routing study, more QoS parameters can be considered to help the routing algorithm select better routing paths based on user QoS needs.

**Table 9:** The summary of the works related to the QoS-based dynamic routings.

Network Structure	Reference	Proposed Algorithm/Scheme	Features/Advantages
MEO–LEO	[72]	A new dynamical hierarchical distributed QoS routing protocol	QoS metric information composed of delay and bandwidth
	[74]	A sustainable heuristic QoS routing algorithm	Utilized heuristic algorithms to optimize routing
	[77]	An adaptive QoS dynamic routing	Collected QoS status between the link from the source node to the destination node, set QoS parameters based on specific satellite scenarios
	[86]	A routing algorithm based on priority and probability	Low-priority services forwarded to MEO satellites, considered both bandwidth and latency QoS parameters
GEO–MEO–LEO	[73]	A multi-QoS optimization routing	Effectively balanced network traffic load, avoided the constraints of onboard network resources
GEO–HEO–LEO	[75]	A QoS-aware transport routing	Build multi-cast trees with the LCT approach
GEO–LEO	[78]	A routing algorithm based on broadband services	Related weights are introduced, improving communication quality
	[79]	A QoS-aware routing scheme	Used the link throughput and end-to-end delay as path cost factors; the GEO and the LEO satellite layer are relatively independent
	[82]	A QoS routing scheme with weights	Adopted feature vector to set weights for QoS state values, balanced the network traffic
DTN-deployed satellite networks	[80]	A new QoS-enabled routing strategy	Utilized STAG to implement a routing scheme
STAG-based mission model	[81]	A QoS multiple routing algorithm	Considered QoS for multiple transport tasks; flow and latency requirements are accounted for
BRSN reputation model	[83]	A dynamic QoS routing scheme	Optimized heuristics from a holistic perspective, scheme included route lookup and routing table update

The QoS-based dynamic routing algorithm studied above mainly considers some traditional QoS parameters, including the available bandwidth, path delay, packet loss rate, and other parameters of available resource allocation. However, these routing algorithms fail to provide trusted routes when there is an attack on the network. The SDN framework has emerged as a promising QoS-assurance solution. The multi-layer satellite network model with SDN architecture meets QoS requirements and outperforms others in end-to-end delay, packet loss rate, and network throughput. In 2020, based on hte trusted resource matrix (TRM), the work in [87] presented a trusted routing (TR) model to protect normal ISTN traffic from network attacks. In the process, the SDN controller constructs the feature matrix by collecting the state information of each routing node. An entropy estimation method is used to determine if a routing node is under attack. The scheme discovers trusted transmission paths through the TR model to assign trust values to TRM routing nodes. Based on careful consideration of the available and trusted resources, a hybrid routing model (HR) was proposed. The model introduces TRM into the QoS evaluation system and selects trusted paths to avoid attack traffic. The HR algorithm combines QoS and transport requirements to provide trusted routes. In the simulation experiments, the proposed algorithm is compared with the traditional QoS-based routing (QR) algorithm and the shortest path (SP) algorithm. The results show that the TR algorithm and the HR algorithm can avoid network attack traffic better and maximize the security of normal traffic. Furthermore, the HR algorithm can utilize limited resources better than the TR algorithm. However, the hybrid routing model fails to optimize the distribution of weights between available and trusted resources. There may be an optimal weight ratio based on network states. The HR model is better for trusted path selection and packet transmission efficiency. The issue will be addressed in the following research and analysis.

In wireless networks, scheduling algorithms are a prominent topic, notably on GEO satellites that employ digital video broadcasting satellite second-generation (DVB-S2). Designing a proper scheduling method is crucial for GEO satellites in a multi-layer satellite network to meet QoS limitations. Therefore, the work in [88] developed a hierarchical scheduler to support different QoS levels for user traffic while avoiding performance degradation. In the literature [89], the authors designed a cross-layer packet scheduler based on the physical and network layers. Simulation results show that this scheme maximizes bandwidth utilization based on service priority. The work in [90] proposed a two-step scheduling technique to provide fairness, QoS, and performance for satellite terminals. However, these scheduling techniques have two issues. First, the system cannot identify the priority of each QoS. Second, the channel state cannot be monitored. Therefore, a scheduling strategy must provide QoS-based distinction.The packet scheduler can determine the QoS priority depending on the channel status of each target user. To improve the performance of the digital video broadcasting-satellite second generation extension (DVB-S2X) satellite platform, the authors proposed two new two-stage scheduler (TSS)-method scheduling algorithms [91]. The first stage of the TSS uses an adaptive priority scheduler (APS) to meet QoS requirements and better distribute IP packets in the MAC frame queue. An improved version of proportional fair (PF) queuing, called PFPP, is considered in the second stage. The approach decreases packet data, improving the packet drop rate, E2E latency, and delay jitter. The approach uses distinct scheduling pairs in the first and second phases of the scheduler to evaluate the system’s packet loss rate, packet latency and delay jitter. The scheduling scheme improves packet drop rate, delay jitter, and packet delay, according to simulations. In particular, the method balances QoS, link traffic allocation, and throughput in a satellite environment.

#### 4.2.3. Traffic Balancing Dynamic Routings

Traffic-balanced dynamic routing is similarly a key research topic in multi-layer satellite networks. Such routings can enhance the throughput of the entire satellite network and decreases the packet loss rate of the network. As the satellite network topology is predictable and periodic, the satellite can predict traffic congestion and adjust its routing strategy. Table 10 summarizes the works related to the traffic balancing dynamic routings.

The hierarchical QoS routing protocol (HQRP), designed in [92], was the first proposal for multi-layer satellite networks. On this basis, LEO satellites are responsible for modifying routes, while the MEO satellites are distributing routes, thus enabling the rapid upgrading of routes. However, HQRP assumes that the ISLs of the inter-satellite links between LEO layers are independent of each other. Since MEO satellites are charged with the communication of the entire satellite network, there are significant constraints on the operational efficiency of the system. Multi-layer satellite networks can be improved by combining the benefits of satellites at different altitudes. In MLSN, packets were transmitted from the information source to their destination through each node of the space network. Any changes between nodes in the satellite network would affect the flow control of the upper system. Table 11 shows the the layers’ performance factors, technologies, and mechanisms. Therefore, the design of the MLSN should also consider the cross-layer optimization principle and adjust the initial weight of different service links.

Therefore, the work in [93] presented the multi-layered satellite IP Networks algorithm. The scheme employs MEO satellites for LEO topology design and GEO for LEO routing, reducing communication costs and algorithm complexity. However, the dispersed MEO satellites prevent LEO satellite links from receiving real-time dynamic topology information, causing congestion. Distributed scheduling is the best load-balancing strategy for each region. Moreover, the algorithm is only for instantaneous communication for local and adjacent satellite networks. When a group of satellites is subjected to a large amount of traffic simultaneously, it is likely to cause local congestion in the network. Thus, the work in [94] introduced an explicit load balancing (ELB) routing strategy to handle local traffic congestion. In the routing scheme, satellite nodes only obtain the traffic state of their neighbors to route the global network. However, the approach has not explicitly proposed an optimal traffic allocation strategy to avoid link congestion problems, and the ability to solve severe network congestion is greatly limited. Inspired by traffic lights, the congestion status of satellite nodes can be indicated by the status of traffic lights, similarly [95]. While delivering packets on a predetermined path, the satellite link can dynamically adjust the network routing based on the real-time colors of the data center traffic lights. Each packet can eventually obtain a near-optimal transmission path by planning and dynamically adapting routing paths in real-time. The approach takes full advantage of the real-time queuing delay of the satellite link and the congestion between the current and next-hop satellite nodes. The packet loss rate and queuing delay are diminished, and the transmission efficiency of the network is enhanced. However, such a method avoids the link congestion by sending packets to inactive satellites, leading to link congestion on other satellites. The work in [96] suggested a routing protocol to balance delay-sensitive, video application, and other transmission traffic. Latency-sensitive traffic has the highest priority and can be transmitted first when passing over a congested link. Images transmitted on LEO satellites are detoured when passing through congested areas. Other data can be transmitted by GEO satellites. However, Maximum traffic can only go through GEO satellites regardless of network congestion, increasing end-to-end delay. Based on the idea of traffic splitting, the work in [97] dispersed the LEO satellite traffic that occurred congestion to the MEO satellites. By using the idea of an adaptive routing protocol for QoS (ARPQ), calculating the maximum traffic threshold of the link was proposed in [98]. When the traffic transmitted on the link exceeds the threshold, the congested traffic is assigned to the MEO satellite. Otherwise it is transmitted directly to the LEO satellite. ARPQ is a two-layer satellite routing protocol with some congestion control mechanism, but it will cause congestion on satellite links at the LEO and MEO layers. To address this concern, the work in [99] introduced SLSR. SLSR takes into account the latency of the network and the expected waiting delay when calculating routes. Such an approach allows network data traffic to be evenly forwarded over multiple lightly loaded links instead of the shortest link.

To better utilize ISLs and achieve load balancing across the constellation, in 2015, the work in [100] proposed a traffic-load-aware routing (TLAR). The scheme transforms the traffic congestion question into a convex optimization problem. The approach reduces the end-to-end latency of the network by minimizing network traffic while maintaining high throughput. However, since the time-varying network topology is not introduced into the network, the reliability of routing cannot be fully guaranteed when large-scale data transmission is performed in the LEO satellite network. Due to the complex network structure, diverse transmission traffic, and dynamic multi-layer satellite networks, the traditional traffic scheduling algorithm is difficult to model and analyze. In 2017, the work in [101] introduced a new traffic balancing algorithm for LEO–MEO networks. The algorithm exploits multi-path routing algorithms to improve link utilization. By accessing the LEO–MEO network and transmitting data in conjunction with real-time status information about the current link, the transmission queue in the link avoids network congestion. In multi-layer satellite networks, owing to the non-equal distribution of users, LEO satellites are prone to massive network congestion when they pass through areas of high traffic. Moreover, LEO satellites in the same region may share a GEO manager to optimize the topology and congestion control, causing link congestion. In 2018, the work in [102] suggested a new two-layer satellite load-balanced cooperative data transmission scheme. Each MEO satellite manages LEO satellites in groups, calculates routing tables, and collects routing information in the routing scheme. During data transmission, when congestion occurs in the LEO layer, each LEO satellite adaptively selects MEO satellites for data forwarding based on the MEO layer link status report. To a certain extent, the scheme can effectively alleviate the congestion of MEO satellites. The method optimizes the LEO–MEO satellite network topology, addressing MEO traffic congestion. After analyzing the dynamic characteristics of each layer of the satellite, the authors presented a fast topology-based conversion algorithm. The algorithm decreases the topology snapshot switching frequency and improves communication efficiency in satellite networks. In addition, to implement satellite services that satisfy QoS, the author simultaneously proposed a service-blocking-based network management mechanism. However, the traffic balancing mechanism fails to account for the queuing delay in the link. Similarly, the existing dynamic balanced routing for traffic focuses on end-to-end delay. Nevertheless, such routing algorithms perform better only when the traffic is not overloaded. As the complexity of the network increases, multi-layer satellite network service requirements grow, causing a high packet loss rate and long queuing delay. Additionally, as each LEO satellite covers a smaller area, the unbalanced intensity of service demand in multi-layer networks may cause more severe link congestion and longer queuing delays. Therefore, actors such as congestion and queuing delay need to be added when designing the routing scheme for dynamic traffic balancing. Based on the prediction of traffic distribution, the work in [103] presented a parametric adaptive multi-attribute decision making (PASMAD) access and switching algorithm for GEO–LEO heterogeneous satellite networks. The algorithm customizes a dynamic parameter to balance the network load and solve link congestion caused by uneven traffic distribution in heterogeneous networks. In addition, for the improved approach, the throughput of the entire heterogeneous network can be boosted while ensuring the QoS for users. The method fails to optimize the satellite network’s topology and reduces its fast conversion capability to improve communication performance. Furthermore, since the algorithm adopts real-time data collection to complete the routing operation, a corresponding load balancing mechanism is omitted.

As network complexity increases, multi-layer satellite network service requirements grow, causing a high packet loss rate and long queuing delay. Additionally, as each LEO satellite covers a smaller area, the unbalanced intensity of service demand in multi-layer networks may cause more severe link congestion and longer queuing delays. Therefore, congestion and queuing delays must be added to the routing scheme for dynamic traffic balancing. In 2019, the work in [104] investigated a new routing update method. The method accurately detects queuing delays and dynamically adjusts routing paths according to queue changes. The solution can predict link conditions before congestion occurs on the link and reasonably select suitable routes based on various traffic situations in the link. Consequently, all satellites in the network have more possibilities to receive packets from neighboring satellites, solving the traffic congestion issue of the next hop in satellite nodes to some extent. Moreover, in a multi-layer satellite network, multiple satellite nodes need to transmit data simultaneously. However, the limitation of the storage resources of the satellite network can make the data among the nodes conflicting. To better allocate the service resources in the satellite network, the work in [105] adopted a routing method based on game theory and distributed cache negotiation. The approach proposed a new competition model for LEO satellite traffic to maximize the use of traffic resources. Furthermore, Stackelberg traffic balance routing uses a threshold function to convert non-convex optimization into convex optimization. However, the method has limitations to routing and data relaying, which causes transmission delays and packet losses. The balanced dynamic routing of traffic in multi-layer satellite networks requires the ability to predict and analyze end-to-end traffic. In 2020, the work in [106] designed a communication architecture with an energy metering node (EGN) at its core. The scheme developed a unified traffic balancing strategy for resource-limited multi-layer satellite networks. The approach ensures that the network remains operational for the maximum possible time, extending the lifetime of multi-layer satellite networks. However, the algorithm is based on a grid-type satellite network with a static and relatively time-invariant topology.

For multimedia services, user satellites are not consistently grouped in small clusters, so grid-based traffic allocation methods are no longer applicable. Link overload traffic in densely populated areas can easily cause network delays and throughput crashes due to satellites’ geographical limitations. An effective traffic-congestion-aware routing scheme is indispensable to ensure the communication quality of multi-layer satellite networks. Therefore, in 2021, the work in [107] proposed a traffic-balancing strategy based on a hybrid satellite network. In the solution, the SDN controller is employed to evaluate the blocking situation of the link and to ensure the flexibility of the hybrid satellite network simultaneously. The strategy schedules traffic to empty satellite nodes on the same layer for congested link traffic. To a certain extent, it solves the traffic congestion issue in the link. In addition, compared to traditional satellite network traffic-balancing algorithms, the method has a better ability to distribute link traffic. However, such traffic balance routing strategies lack flexibility for multi-layer satellite networks with dynamic structures. Meanwhile, such an algorithm makes it hard to perform accurate modeling and analysis. Proper end-to-end (E2E) traffic prediction in multi-layer satellite networks plays a critical role in balancing traffic. The work in [108] presented an improved Markov model (HMM)-based method for E2E traffic prediction. The GEO satellite is the main controller of the SDN system, and it can obtain some traffic data from the LEO satellite it controls. Owing to the limitation of the number of GEO satellites, the algorithm adapts the idea of gradual convergence. Simulation experiments on the HMM verify the effectiveness of the algorithm. Moreover, despite the large volatility of the actual service traffic in existing multi-layer satellite networks, the approach is still better at predicting and tracking the traffic on the links with low error. However, the proposed scheme fails to consider the dynamic characteristics of the multi-layer satellite network and uses a virtual topology instead. The routing scheme assumes the dynamic network topology is stationary in a given time interval, limiting its use in multi-layer satellite networks. To address the issue, the work in [109] designed a multi-agent reinforcement-learning-based traffic scheduling algorithm for multi-layer satellite networks. With reinforcement learning techniques, the SDN can fully combine the centralized control capability of the SDN and the dynamic adaptive capability of reinforcement learning. By using neural networks to model reinforcement learning and SDNs, the dynamics of network traffic can be accurately sensed, and routing paths can be optimized in a timely manner. The algorithm takes GEO satellites as agents for training. By improving the experience pool in reinforcement learning, it is possible to enable GEO satellites to adapt to changing link states in multi-layer satellite networks continuously. The trained traffic scheduling model can make fast routing decisions for congested traffic detected over the link and maximize link utilization. Additionally, the algorithm deploys SDN controllers on GEO satellites, allowing the monitoring of topology changes and traffic status across the satellite network. Since the research on SDN traffic detection is very mature, the algorithm balances network traffic by detecting congested traffic. Efficient traffic distribution over heterogeneous links in satellite data relay networks (SDRNs) is equally a key concern in current research. However, diverse traffic types and heterogeneous links complicate link delay evaluation, affecting traffic distribution efficiency. Therefore, the work in [110] extended spatial traffic to arbitrary categories and analyzed the effect of the link heterogeneity on link traffic distribution simultaneously. The algorithm introduces a delay upper bound in heterogeneous links to optimize traffic allocation with the least delay. Compared to the inherent characteristics of homogeneous flows, the inherent characteristics of flows within polymerization heterogeneity have been radically changed. Therefore, in future research, for the multi-layer satellite network traffic-balancing dynamic routing algorithm, how to achieve the optimal traffic distribution of heterogeneous links will become the focus of attention of academia and industry. Comparisons of the key performance of partial dynamic routings in multi-layer satellite networks are shown in Table 12.

**Table 10:** The summary of the works related to the traffic-balancing dynamic routings.

Network Structure	Reference	Proposed Algorithm/Scheme	Main Contributions
GEO-LEO	[96]	A balanced traffic routing scheme	Delay-sensitive traffic can be prioritized for transmission, the maximum flow is only through the GEO satellite
	[103]	A parametric adaptive multi-attribute decision making access and switching algorithm	Load traffic balancing can be achieved by adjusting dynamic parameters
	[105]	A routing method based on game theory and distributed cache negotiation	A new competition model for LEO satellite traffic is proposed to convert non-convex issues into convex optimal solutions
	[108]	An improved Markov-model-based E2E traffic forecasting method	Adopted the idea of gradual convergence
	[109]	A traffic scheduling algorithm based on multi-agent reinforcement learning	SDN and RL are utilized, utilized neural network modeling, deployed SDN controllers on GEO satellites
MEO–LEO	[97]	A delay-based traffic distribution scheme	Sent congested LEO satellite traffic to MEO satellite
	[98]	A traffic distribution scheme	The maximum traffic threshold of the link is calculated, allocated overload traffic to MEO satellites
	[100]	A traffic-aware routing scheme	Convert the flow problem into a convex optimization issue, maintain high throughput
	[101]	A new traffic-balancing algorithm	Improve link utilization with multi-path routing algorithms, data transmission based on the real-time status of the link
	[102]	Load-balanced transmission scheme	MEO satellites managed LEO satellites, calculated routing tables, and collected routing information
Heterogeneous satellite networks	[106]	An efficient load-balancing scheme	Static and relatively time-invariant grid topology
SDN-SAG	[107]	A congestion-aware routing scheme	SDN controller evaluated the blocking of the link, allocated traffic to idle satellite nodes
Satellite data relay networks	[110]	A traffic balanced routing algorithm	Extend traffic to any type, launched upper bound on delay in heterogeneous links

**Table 11:** Performance in different layers.

Index	Physical Layer	Datalink Layer	Network Layer
Delay	Signal delay, propagation delay	Handover delay, congestion control, retransmission protocol	Handover delay, IP mobility management, routing algorithm
Reliability	Channel conditions, path loss, error rate, interference	Forwarding mechanism error	Topological structure
Energy efficiency	Power control, channel conditions, interference	Length control, packet retransmission count	Routing protocol

**Table 12:** Key performance considered by partial dynamic routings in multi-layer satellite networks.

Scheme	Time Delay	Packet Loss Rate	Calculation Overhead	Throughput
ARSA-routing [59]	✕	✓	✓	✕
SDN-MPTCP [60]	✕	✕	✓	✓
QSR, QBA [61]	✓	✕	✓	✕
ASRS [64]	✕	✕	✓	✕
MPRA [68]	✓	✕	✓	✕
QoSRA [69]	✓	✓	✕	✓
FCMR [70]	✓	✕	✕	✓
HDRP [71]	✓	✕	✕	✕
Multi-QoS routing [72]	✓	✕	✕	✓
Heuristic QoS routing [73]	✕	✓	✕	✓
SADR [76]	✓	✕	✓	✕
Bandwidth-routing [77]	✓	✕	✕	✓
Admission Control routing [78]	✓	✕	✕	✕
STAG routing [79]	✓	✕	✕	✓
QSMR [80]	✓	✕	✕	✓
Multi-constraint QoS routing algorithm [83]	✓	✓	✕	✕
TDRP [84]	✕	✕	✓	✕
Priority-based routing algorithm [85]	✓	✓	✕	✓
TLAR [99]	✓	✕	✕	✓
PASMAD [97]	✓	✕	✕	✓
Queue State routing [103]	✓	✕	✕	✕
Load-balanced routing [104]	✕	✕	✓	✓

## 5. Potential Technologies and Future Directions

Due to cyclical changes in satellite network topology, designing routing technology has always been challenging. Although, there are a variety of multi-layer satellite network routing protocols and algorithms, as previously described, systematic routing algorithms are still lacking. This section discusses the potential technologies and the future directions of multi-layer satellite networks according to the existing works.

### 5.1. Potential Technologies

According to the current research on multi-layer satellite networks and the results of existing works on dynamic routing mentioned, some potential technologies and directions for future dynamic routings in satellite networks and space–air–ground integrated network are provided.

**Figure 10:**
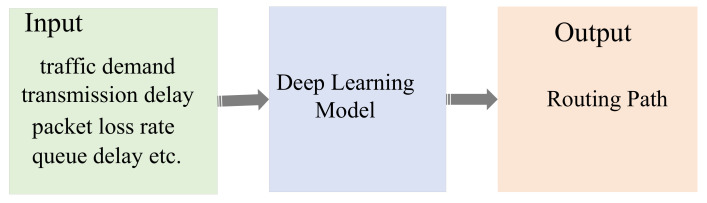
Deep-learning-based routing model.

**Machine Learning (ML):** The dynamic network topology increases the complexity of satellite networks, and link switches in satellite networks exacerbate routing design problems. In recent years, academics have tried to use machine learning to optimize satellite routing [111]. Artificial intelligence (AI) has a strong learning ability and good generalization, making an intelligent network layer possible. In contrast to traditional mathematical model-driven distributed routing algorithms, machine-learning-based routing algorithms are usually data-driven, allowing them to adapt better to changing satellite networks and optimize network performance metrics dynamically. By using deep learning, significant progress has been made in regulating congestion at the transport layer [112], detecting network security [113], and optimizing video stream transmission [114]. In particular, Figure 10 shows common deep-reinforcement-learning-based routing solution models for traffic prediction routing optimization problems [115]. Handling routing tables generated by different layers of satellite nodes in a satellite network is an important challenge in the satellite routing optimization problem. To alleviate the computational overhead of processing the data, ML can be considered to improve the data efficiency of the routing training process, especially for multilayer satellite networks. To further exploit the topology information, the work in [116] designed a distributed intelligent routing algorithm based on GRU and GNN. The trained GNN model is able to achieve 98% accuracy within 15 iterations relative to shortest-path routing.

However, the high input/output feature dimensionality can be formulated via objective function variables, and the satellite routing optimization problem can be formulated via objective functions. The satellite routing optimization problems are generally complicated to solve via traditional methods. Machine learning methods seem to have a much better performance in solving this kind of optimization problem, according to the existing works in the academic literature.

**Mobile Edge Computing:** Edge computing enables these resources at the edge of the network to meet critical industry needs for agile connectivity, real-time services, data optimization, application intelligence, security, and privacy [117]. New service models such as mobile edge computing (MEC) and fog computing architectures break the obsolete and limited framework of the cloud computing system [118]. They bring computing and storage resources closer to the devices. So, the high computing agility and low latency can be achieved [119,120]. Compared to traditional cloud computing models, edge computing models have the merits of real-time data processing and analysis, high security, privacy protection, scalability, location awareness, and low traffic.

Dynamic topology policies send data packets between satellite nodes and use real-time routing information to choose the best routes in satellite networks. However, the dynamic routing mechanism requires a high level of data processing due to the satellite’s motion. There is no doubt that edge computing technology with powerful data processing and analysis can solve this problem. Mobile devices can collect and analyze vast amounts of data, crucial for satellite operations. Implementing the deployment of edge servers near mobile devices can enhance data analytics through high network bandwidth and low latency.On-board processing technology enables multi-layer satellite networks with more powerful communication abilities, equipped with the MEC server [121,122,123]. Hence, for the giant multi-layer satellite networks, more attention should be paid to the cooperation of mobile edge computing.

**Digital Twin:** Digital twins are physical models created digitally to analyze and optimize physical objects [124,125,126]. The technology is supposed to have the characteristics of scalability, interoperability, scalability, and fidelity [127,128].

Based on the digital twin satellite network model and simulation technology, a dynamic topology model of multi-layer satellite networks is constructed in the information space to predict and simulate dynamic routing changes. On the one hand, the prediction results of the simulation are applied to select routings and forward decisions. On the other hand, the large amount of data generated by monitoring the solid satellite platform is used to improve the accuracy and predictability of information space models., significantly reducing the computational complexity of the dynamic routing strategies in multi-layer satellite networks.

DT can reproduce the operational status of satellites using available data to assist decision making, prevent anomalies and problems from worsening, and improve satellite failure detection. In addition, during content delivery, virtual routing in digital twin networks (DTNs) can be used to calculate and verify optimal routing paths [129,130].

### 5.2. Future Directions

Due to the substantial heterogeneity of multi-layer satellite networks, the frequent changes in the constellation topology, and inter-satellite links, flexible control system reconfiguration is more necessary. The new research direction of routing will undoubtedly become a hotspot in future academic work. Its primary contents are as follows:

**Multi-scale Information Awareness and Computing In Complex Environments:** The satellite networks contain multiple types of network nodes, and the data acquired by the network nodes in different satellite layers are heterogeneous. To facilitate the processing of massive data information, we must consider unifying and integrating data when designing routing algorithms. In order not to waste many satellite network resources in terms of bandwidth, mobile edge computing (MEC) platforms can be used for big data analysis [131]. Big data analytics can be performed at the edge of the network, and the results are sent to the core network. MEC platforms allow edge networks to gain management access to computing and services to reduce mobile users’ network latency and bandwidth consumption. Content optimization is indispensable to better support routing requests that differentiate the quality of service. With MEC, content optimization can be performed dynamically based on user-perceived information. At the same time, edge networks can improve performance, enhance the quality of user experience, and provide new services through content optimization. Furthermore, the use of MEC can improve the routing algorithm’s ability to sense the network state. When the network state changes, the algorithm quickly converges and makes a better routing decision. The field of autonomous driving, where edge computing and deep learning are closely integrated, has been carefully studied by several researchers in China and abroad [132,133].

In the subsequent research, deep learning and reinforcement learning can be used to improve satellite network routing decision making. In particular, improving deep learning technology and expanding MEC’s use in satellite networks to boost network performance will be important.

**Intelligent Satellite:** An intelligent satellite is an operating system with strong fault tolerance running on a standardized hardware platform. Various types of apps can be developed to carry out satellite applications based on this operating system. Smart satellites have the following typical characteristics: (1) Requirements are definable. The satellite can reconfigure its entire system and respond flexibly to different space mission needs, such as communication, navigation, remote sensing, and scientific exploration, providing a variety of functions and tasks; (2) Software can be reconfigured. The satellite has a consistent program execution environment and a rich set of application software that can dynamically configure and execute apps for different tasks; (3) Reconfigurable functionality. Different functions can be quickly reconfigured by accessing different hardware components and loading different software components.

Inter-layer contacts are frequently interrupted in multi-layer satellite networks due to the rapid relative motion between satellites in different layers. The network topology is constantly changing, leading to a frequent dynamic reconfiguration of inter-satellite routes. Hence, building a multi-layer satellite network based on artificial intelligence is the way forward, such as intelligent seamless handoff technology and intelligent interference cancellation technology to respond to dynamic routing effectively. To some extent, we can achieve intelligent communication. In addition, artificial intelligence will become an inherent feature of future satellite networks, including intelligent network elements and network architectures, intelligent connected objects, and intelligent information support services.

**Intelligent Routing:** Intelligent routing has three advantages [134]: (1) Accuracy. The ML algorithm model is trained without complex assumptions and network modeling; (2) Efficiency. Fast inference based on the input data can obtain the optimized routing decision in polynomial time; (3) Generality. The same ML model can be used to solve different network optimization problems depending on the training data. In the next satellite routing optimization matter, scalability is an important property to satisfy the routing algorithm. Existing ML routing algorithms handle small topological networks with up to 20 satellite nodes. A larger topology means an exponentially growing number of network states and a greater difficulty in routing decisions. Future intelligent routing algorithms must achieve good results on large topologies. Compared with traditional routing algorithms, intelligent routing requires more computational resources and higher routing performance. In addition, for smart routing, how to deploy in real scenarios is also a great challenge. Although SDN networks have enhanced the computational power of the router control layer, intelligent routing algorithms are still difficult to deploy at scale in existing satellite network architectures [135]. In the future, smart routing algorithms may focus on designing smart routing devices.

## 6. Conclusions

This paper provides a comprehensive overview of the existing dynamic routing schemes for satellite networks in academia. First, satellite network characteristics are introduced, and essential dynamic routing technologies and challenges are evaluated. Due to the constraints of single-layer satellite dynamic routing, this paper focuses on SDN-based, QoS-based, and traffic-balancing multi-layer satellite dynamic routings. In addition, to better optimize the performance of future dynamic routing schemes for satellite networks, especially for multi-layer networks, potential technologies such as ML, MEC, and Digital Twin are also discussed in this paper. Finally, this paper predicts the future development trends of dynamic routing methods in satellite networks, such as multi-scale information sensing and processing in complicated settings, intelligent satellites, and intelligent routing.

## Figures and Tables

**Table 1:** Comparison of the partial algorithms.

Routing	Architecture	Description	Characteristics
LZDR	Single-Layer	Connection-oriented routing algorithm based on virtual nodes	Dividing routing based on neighboring virtual nodes. Reduce communication overhead by overlay routing.
DRA		Typical virtual node routing algorithm	Routing is performed by logical position. Source satellites have rerouting capabilities
LCPR		An advanced DRA routing algorithm	Optimal paths are obtained utilizing distributed computing methods
FHRP		Connection-oriented routing algorithm	Effectively simplifies routing and minimizes routing overhead
LRES		Load-balanced routing algorithm	Dynamically adjusts routes based on link status Effectively prevents network congestion
SGGM	Multi-layer	Group-managed routing algorithm	Forwarding of packets and calculation of routing tables is independent of mutually.
DDRA		Data-driven routing algorithm	Provides optimal routing Better network adaptation.
SGRP		Satellite grouping and routing protocol	Provides a mechanism to resolve congestion and satellite failures.
HSRP		Hierarchical satellite routing protocol	Improves network resource utilization.
ERRS		Expanding Range Route Selection	Reduces end-to-end latency and increase network throughput.

**Table 2:** Comparison of related works.

Network Type	Reference	Feature	Main Contributions
SAGSIN	[14]	Integrated	Reviewed the architecture and key technologies of SAGINs and summarized potential technical challenges of SAGIM
SAGIN	[8]		Studied traffic-based algorithms and quality of service (QoS)-based IP routing algorithms Revised several critical factors of the genetic algorithm
	[13]		Reviewed the architecture and key technologies of SAGINs and summarized potential technical challenges of SAGIM
	[17]		Designed a SAGIN framework and a deep-reinforcement-learning-assisted routing algorithm
	[10]	Multi-layer	Summarized routing methods in multi-layer satellite networks
Satellite Networks	[15]	Singer-layer	Summarized the main issues faced by large LEO satellite networks and analyzed the main directions for the future development of the network
	[9]		Reviewed the routing algorithms based on virtual nodes in single-layer satellite networks
	[11]		Summarized the development routing algorithms for single-layer satellite networks
	[12]		Investigated a partial load balanced routing algorithm based on link state
	[16]		Investigated a deep-reinforcement-learning-based routing scheme for satellite routing

**Table 3:** Comparison of subnetworks.

Architecture	Network Scenario	Control Pattern	Performance	Limitations
MEO-LEO	Satellite	Central	Short delay, low power requirements	Dynamic Inter-satellite Links
GEO-LEO	Satellite-terrestrial	Central	Large network coverage, and high on-star processing power	Dynamic link connection, and high implementation costs
GEO-MEO-LEO	Satellite	Central	Robust link and superior coverage performance	High construction and maintenance costs, difficulty of implement

**Table 5:** The summary of the works related to the virtual nodes routing algorithms.

Proposed Algorithm/Scheme	Reference	Features/Advantages	Results
A distributed routing algorithm	[34]	Divided the surface space into several specific spaces with corresponding logical areas	Avoided dynamics with satellite nodes in the logical region
A priority-based adjustable routing, enhanced priority-based adjustable routing	[35]	Balanced load with link utilization and historical data	Reduced redundant traffic data and better utilization of ISLs
A virtual node switching algorithm, multi-state virtual network switching algorithm	[36]	Increased number of satellites per orbit to enable soft switching	Reduced data loss and latency
A dynamic detection routing scheme	[37]	Lower snapshot latency and fewer path changes	Improved network stability and adaptive capabilities, increased routing computation overhead for the network
A load-balancing routing algorithm	[38]	Applied distributed computing to optimize paths	Saved large amounts of on-star computing resources, reduced average queue latency and packet loss
A probabilistic routing scheme	[39]	Based on the latitude and longitude of the satellite nodes to select the routing path	Reduced time complexity, achieved load balancing of services
A local repair-based, disruption-resistant, on-demand routing scheme	[40]	Applied local repair strategy	Enhanced the real-time performance of the network

**Table 7:** Performance comparison of partial single-layer satellite routings.

Scheme	Time Delay	Packet Loss Rate	Calculation Overhead	Throughput
Unicast routing [27]	✓	✕	✕	✓
Geographical routing [28]	✓	✕	✓	✕
Broadcast routing [31]	✕	✓	✓	✓
MAC-routing [32]	✓	✕	✓	✕
MSVN-SHO, VN-HO [36]	✓	✓	✕	✕
DDRA [37]	✓	✓	✕	✕
LCRA [38]	✓	✓	✕	✓
LCPR [39]	✕	✓	✕	✓
DODR [40]	✓	✓	✕	✕
CEMR [46]	✓	✕	✕	✓
DTBR [50]	✕	✕	✓	✓
DFS [51]	✕	✕	✓	✓
MA-routing [52]	✓	✓	✕	✕
DCCR [53]	✓	✕	✕	✓

## Data Availability

Not applicable.

## Abbreviations

The following abbreviations are used in this manuscript:

GEO	Geosynchronous earth orbit
SIN	Space information networks
LEO	Low-Earth orbit
STAG	Storage time aggregated graph
MEO	Medium-Earth orbit DTN
DTN	Disruption-tolerant networks
QoS	Quality of services
TNM	Time-domain grid model
TLR	Traffic light-based intelligent routing
SSNs	Small satellite networks
IP	Internet protocol
DRLR	Deep-reinforcement-learning-based routing algorithm
VANTs	Vehicular and ad hoc networks
FRA	Fast response anti-jamming algorithm
UAVs	Uncrewed aerial vehicles
DRSA	Dynamic routing algorithms
LZDR	Localized zone distributed routing
GCR	Contact graph routing
LCPR	Low-complexity probability routing algorithm
ERRA	Extended range routing algorithm
DRA	Driven routing algorithm
DRA	Distributed routing algorithm
SGGM	Satellite group and group management
PAR	Priority-based adjustable routing
DTN	Disruption-ant network
E-PAR	Enhanced Priority-based adjustable routing
DDRA	Data-driven routing algorithm
VN	Virtual node
DVTR	Dynamic virtual topology routing
VN-HO	Virtual node handover operating
FHRP	Footprint handover rerouting protocol
MSVN	Multi-state virtual network
LRES	Inter-satellite link congestion
DDRA	Dynamic detection routing algorithm
ISLs	Inter-satellite links
LCRA	Low complexity routing algorithm
SAGIN	Space–air–ground integrated network
DSP	Dijkstra’s shortest path
IM	Immersive media
DODR	Disruption-resistant on-demand routing
SCN	Service customized network
SAG-IM	SAGIN architecture for IM
LAOR	Location-assisted on-demand routing
AODV	Ad hoc on-demand distance vector
SAGSIN	Space–air–ground–sea integrated network
LDPC	Low-density parity check
SDN	Software-defined network
DV-DVTR	Dynamic virtual topology routing
MPLS	Multiprotocol label switching
DTVTS	Discrete-time virtual topology setup
OSPF	Open shortest path first
DTPSS	Discrete-time path sequence selection
LDP	Label distribution protocol
FSA	Finite state automata
UDL	Up/down link
CEMR	Compact explicit multi-path routing
IM/DD	Intensity modulation and direct detection
MSVN	Multi-state virtual network
OBP	Onboard processing
MSVN-SHO	MSVN-based satellite networks
OBS	Onboard switching
VN-HO	Virtual node handover
OBR	Onboard routing
DTBR	Distributed traffic-balancing routing protocol
SDSN	Software defined software defined satellite networks
MPTCP	Multi-path transmission control protocol
SERvICE	Satellite-ground satellite communication networks
QSR	QoS-oriented satellite routing
ARA	Adaptive Routing Algorithm
DDPG	Deep deterministic policy gradient
LSTM	Long short-term memory
NOCC	Network controller control center
ONOS	Open Network Operating System
MPRA	Multi-path routing algorithm
OMHS	Optimal minimum switching strategy
FCMR	Fuzzy CNN-based multi-task routing
GCC	Ground computing center
HDRP	Hierarchically distributed QoS routing protocol
QAMRP	QoS-aware multi-point transport routing
TDRP	Time division routing protocol
TRM	Trusted resource matrix
TR	Trusted routing
HQRP	Hierarchical QoS routing protocol
ELB	Explicit load balancing
ARPQ	Adaptive routing protocol for QoS
TLAR	Traffic-load-aware Routing
PASMAD	Parametric adaptive multi-attribute decision making
ML	Machine learning
MEC	Mobile edge computing
DTNs	Digital twin networks
AI	Artificial intelligence
SLSNs	Single-layer satellite networks
MLSNs	Multi-layer satellite networks
MIA	Multi-scale information awareness
DVB-S2	Digital video-broadcasting satellite—second-generation
DVB-S2X	Digital video-broadcasting satellite—second generation extension
TSS	Two-stage scheduler
APS	Adaptive priority scheduler
